# An open‐source LED lamp for use with the LI‐6800 photosynthesis system

**DOI:** 10.1002/aps3.11622

**Published:** 2024-10-25

**Authors:** Aarón I. Vélez‐Ramírez, Juan de Dios Moreno, Uriel G. Pérez‐Guerrero, Antonio M. Juarez, Hector Castillo‐Arriaga, Josefina Vázquez‐Medrano, Ilane Hernández‐Morales

**Affiliations:** ^1^ Laboratorio de Ciencias Agrogenómicas, Escuela Nacional de Estudios Superiores Unidad León Universidad Nacional Autónoma de México Boulevard UNAM 2011 León 37684 Guanajuato Mexico; ^2^ Laboratorio Nacional PlanTECC, Escuela Nacional de Estudios Superiores Unidad León Universidad Nacional Autónoma de México Boulevard UNAM 2011 León 37684 Guanajuato Mexico; ^3^ Laboratorio Interdisciplinario de Sostenibilidad, Escuela Nacional de Estudios Superiores Unidad León Universidad Nacional Autónoma de México Boulevard UNAM 2011 León 37684 Guanajuato Mexico; ^4^ Instituto de Ciencias Físicas Universidad Nacional Autónoma de México PO Box 48‐3 Cuernavaca 62251 Morelos Mexico; ^5^ Laboratorio de Fisiología Vegetal, UBIPRO, Facultad de Estudios Superiores Iztacala Universidad Nacional Autónoma de México Avenida de los Barrios 1 Tlalnepantla 54090 Estado de México Mexico

**Keywords:** A‐PPFD, actinic light, assimilation rate, LI‐6800, light‐emitting diode, LEDs, photosynthetic light‐response curve, photosynthesis

## Abstract

**Premise:**

Controlling light flux density during carbon dioxide assimilation measurements is essential in photosynthesis research. Commercial lamps are expensive and are based on monochromatic light‐emitting diodes (LEDs), which deviate significantly in their spectral distribution compared to sunlight.

**Methods and Results:**

Using LED‐emitted white light with a color temperature similar to sunlight, we developed a cost‐effective lamp compatible with the LI‐6800 Portable Photosynthesis System. When coupled with customized software, the lamp can be controlled via the LI‐6800 console by a user or Python scripts. Testing and calibration show that the lamp meets the quality needed to estimate photosynthesis parameters.

**Conclusions:**

The lamp can be built using a basic electronics lab and a 3D printer. Calibration instructions are supplied and only require equipment commonly available at plant science laboratories. The lamp is a cost‐effective alternative to perform photosynthesis research coupled with the popular LI‐6800 photosynthesis measuring system.

## Resumen

**Premisa:**

Controlar la densidad del flujo luminoso durante las mediciones de asimilación de dióxido de carbono es esencial para la investigación en fotosíntesis. Las lámparas comerciales son costosas y se basan en diodos emisores de luz (LED) monocromáticos, que se desvían significativamente en su distribución espectral en comparación con la luz solar.

**Métodos y Resultados:**

Utilizando luz blanca emitida por un LED con una temperatura de color similar a la luz solar, desarrollamos una lámpara accesible y compatible con el Sistema de Fotosíntesis Portátil LI‐6800. Cuando se combina con software personalizado, la lámpara puede ser controlada a través de la consola del LI‐6800, por un usuario o mediante código de Python. Las pruebas y calibraciones demuestran que la lámpara cumple con la calidad necesaria para estimar parámetros de fotosíntesis.

**Conclusiones:**

La lámpara puede ser construida utilizando un laboratorio básico de electrónica y una impresora 3D. Se proporcionan instrucciones de calibración que solo requieren equipo comúnmente disponible en laboratorios de ciencias vegetales. La lámpara es una alternativa rentable para realizar investigación en fotosíntesis junto con el popular sistema de medición de fotosíntesis LI‐6800.

A common method to study photosynthesis in vivo requires measuring the net assimilation rate (A) under various environmental conditions, which are carefully set and controlled (Coe and Lin, 2018; Walker et al., 2018; Busch et al., 2024). While ambient carbon dioxide (CO_2_) and irradiance (I) are changed, A is measured, resulting in two dose‐response curves. The first curve is known as the A‐Ci curve, as changes in ambient CO_2_ result in changes in the intracellular CO_2_ concentration (Ci), and the second curve is known as the A‐I curve (Coe and Lin, 2018; Busch et al., 2024). In the International System of Units, I is measured in W∙m^−2^ (J∙m^−2^∙s^−1^); in plant sciences, however, quantum units are preferred, as A increases in response to the number of photons (measured in μmol) with wavelengths between 400 and 700 nm, regardless of their energy content (measured in joules) (Wayne, 2016; Stanghellini et al., 2019). Here, therefore, we measure the light flux density in quantum units (μmol∙m^−2^∙s^−1^) and refer to this as photosynthetically active photon flux density (PPFD), resulting in A‐PPFD curves. The measurements of one or both dose‐response curves can be used to estimate photosynthetic parameters (Yin et al., 2009; Velez‐Ramirez et al., 2014; Busch et al., 2024). As reviewed by Busch et al. (2024), each curve is useful to estimate a distinct set of parameters; for example, an A‐PPFD curve can be used to estimate parameters such as the maximum CO_2_ assimilation rate (*A*_sat_) and the rate of respiratory CO_2_ release from the mitochondria (*R*_d_). The photosynthetic parameters are numeric representations of subprocesses that together contribute to A (Coe and Lin, 2018; Busch et al., 2024). The most common model used to calculate the photosynthetic parameters is the one proposed by Farquhar et al. (1980).

Measuring dose‐response curves requires a way to precisely control the incidence of PPFD on the leaf sample (Coe and Lin, 2018; Busch et al., 2024). Manufacturers of photosynthesis measuring systems typically use lamps based on light‐emitting diodes (LEDs). For example, the LI‐6400 and LI‐6800 Portable Photosynthesis Systems from LI‐COR Biosciences (Lincoln, Nebraska, USA) include LED lamps as optional accessories. Some lamp models, like the LI‐6800‐03, are equipped with blue‐, green‐, red‐, and white‐light LEDs, allowing for fine‐tuning of both intensity and spectrum during measurements. Other manufacturers, such as ADC BioScientific (Hoddesdon, United Kingdom), PP Systems International (Amesbury, Massachusetts, USA), and Heinz Walz (Effeltrich, Germany), also offer lamps equipped with white‐light LEDs for their photosynthesis measuring systems. However, these lamps can be quite expensive due to their carefully engineered designs. This high cost highlights the need for a more affordable, open‐source alternative. In this report, we present the development of such a lamp designed for use with the LI‐6800 photosynthesis measurement system.

A major design goal was to make the lamp compatible with script‐based measurements, that is, to fully integrate the proposed lamp with the LI‐6800 background programs (BP), which are Python scripts that autonomously perform complex measurement procedures (e.g., dose‐response curves). We achieved this integration by using a custom‐made LED driver, the LI‐6800 *User I/O* port, and an Arduino‐compatible (https://www.arduino.cc) microcontroller board, programmed with Python. Hardware, software, detailed building instructions, and calibration procedures are provided under open licenses.

## METHODS AND RESULTS

### Lamp hardware

#### COB‐LED and LED driver

The lamp described here uses a chip‐on‐board LED (COB‐LED), which consists of an array of LEDs tightly packed in a small area. The selected COB‐LED emits white light with a color temperature of 6500 K (see model in Table 1). The COB‐LED array has a forward voltage of 18 V and can operate at a current of up to 1400 mA. In this design, however, its maximal operating current is set at 1000 mA. Thus, the maximal power consumed by the COB‐LED is 18 W.

**Table 1:** List of parts and materials needed to build the proposed LED lamp.

Part	Manufacturer	Model	Quantity needed
Cable, 6 conductors, 24 AWG	Steren, Mexico City, Mexico	M‐06X24MMD‐305 VTA	1.5 m
Cable gland	Heyco, Toms River, New Jersey, USA	M3445	1
Capacitor, 2.2 μF	KEMET Electronics Corporation, Fort Lauderdale, Florida, USA	C322C225K5R5TA	1
Chip‐on‐board light‐emitting diode (COB‐LED) array	CreeLED, Durham, North Carolina, USA	CXB1310‐0000‐000F0HN265E	1
COB‐LED holder	Molex, Lisle, Illinois, USA	180555‐0002	1
CPU cooler, fan, and heatsink	NIDEC, Kyoto, Japan	E97379‐003	1
Diffuser, engineered, 20° square pattern (optional)	Thorlabs, Newton, New Jersey, USA	ED1‐S20	1
D‐Sub standard connector, 25 positions	Purenitetech, Cupertino, California, USA	EZSync906	1
Heatsink, TO‐220	Steren, Mexico City, Mexico	TO‐300	2
M2.5 × 10 mm Pan Head Phillips Screws	McMaster‐Carr Supply Company, Santa Fe Springs, California, USA	92005A071	6
M3 nuts	McMaster‐Carr Supply Company, Santa Fe Springs, California, USA	90593A001	2
M3 socket head screws	McMaster‐Carr Supply Company, Santa Fe Springs, California, USA	M3 × 6 mm (91290A111) M3 × 10 mm (91290A115) M3 × 12 mm (91290A117) M3 × 16 mm (91290A120)	2 6 4 4
M3 washer, nylon	McMaster‐Carr Supply Company, Santa Fe Springs, California, USA	95610A130	2
Microcontroller board, Trinket M0	Adafruit Industries, New York, New York, USA	3500	1
Mirror, glass	Generic	3 mm thick, custom cut	4
Paint	Rust‐Oleum Corporation, Vernon Hills, Illinois, USA	White (7881830) Blue (262156)	1 1
P‐channel MOSFET	ON Semiconductor Corporation, Scottsdale, Arizona, USA	FQPF9P25	1
Precision voltage to current converter/transmitter	Texas Instruments, Dallas, Texas, USA	XTR110KP	1
Power jack	CUI Devices, Lake Oswego, Oregon, USA	PJ‐011A	1
Power supply, AC/DC 24 V	Steren, Mexico City, Mexico	ELI‐2024	1
Power supply, DC/DC 12 V	Monolithic Power Systems, Kirkland, Washington, USA	mEZD72401A‐H	1
Protoboard, 45 × 45 mm	Steren, Mexico City, Mexico	155	1
Resin, SLA 3D printing	Formlabs, Somerville, Massachusetts, USA	FLGPBL01 (Alternative, stiffer resins: RS‐F2‐RG10‐01 and RS‐F2‐RGWH‐01)	500 mL (approx.)
Resistor, Power/Sense	Vishay Intertechnology, Malvern, Pennsylvania, USA	Y09261R00000B0L	1
Resistors	Vishay Intertechnology, Malvern, Pennsylvania, USA	CMF Industrial Series, diverse resistance values	3
Super glue, rubber toughened ethyl cyanoacrylate adhesive	Henkel, Düsseldorf, Germany	Loctite 380	1
Tape, Kapton (polyimide)	McMaster‐Carr Supply Company, Santa Fe Springs, California, USA	7648A733	1 m
Tape, red double‐sided tape for cell phone screen repair	Generic	3 mm wide	1 m
Temperature sensor	Amphenol Corporation, Wallingford, Connecticut, USA	TK95F103W	1
Terminal block, 6 positions	Phoenix Contact, Blomberg, Germany	1725698	1
Thermal paste	Thermaltake Technology Corporation, New Taipei City, Taiwan	TG‐7	1
Thermal pad, dielectric	Wakefield Thermal Solutions, Nashua, New Hampshire, USA	CD‐02‐05‐220‐N	2
Threaded brass inserts	McMaster‐Carr Supply Company, Santa Fe Springs, California, USA	94180A331	10
Trim pot, 100 kΩ	Bourns, Riverside, California, USA	3296W‐1‐104LF	1
Wire, 22 AWG	Alpha Wire, Elizabeth, New Jersey, USA	6823 XX005	1 m
Zenner diode	Vishay Intertechnology, Malvern, Pennsylvania, USA	BZX55B3V3‐TR	1

*Note*: AWG = American wire gauge.

**Figure 1:**
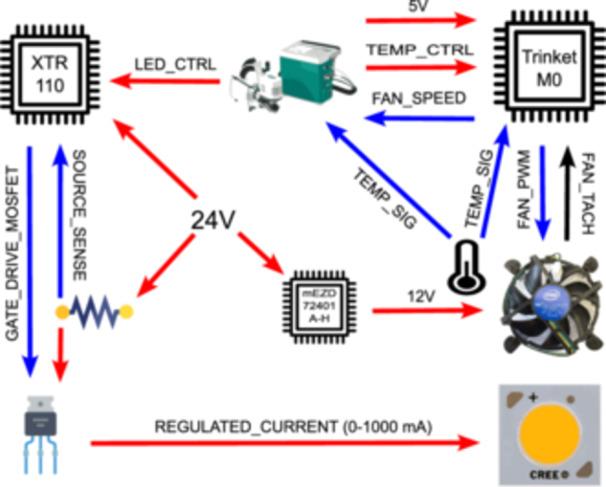
A diagram showing the simplified electronic design of the lamp for the LI‐6800 photosynthesis system. The chip‐on‐board light‐emitting diode (COB‐LED) current is controlled by the XTR110 chip, the fan speed is controlled by the Trinket M0 microcontroller board in response to heatsink temperature, and the COB‐LED current and heatsink temperature setpoints are set by the LI‐6800 via analog signals. Both the LI‐6800 and the Trinket M0 monitor heatsink temperature using a negative temperature coefficient (NTC) thermistor. The Trinket M0 measures fan speed by monitoring the fan tachometer signal (FAN_TACH); then, the Trinket M0 translates the calculated fan speed into an analog signal and sends it to the LI‐6800. An external 24‐V AC‐DC power supply powers an mEZD72401A‐H DC‐DC voltage converter, which in turn powers the XTR110 chip, COB‐LED, and fan with 12 V. Red arrows indicate power sources; black arrows indicate digital signals, and blue arrows indicate analog signals. The complete electronic schematics is available in the GitHub repository (see Data Availability Statement).

The current flowing through the COB‐LED array is regulated by a custom‐designed LED driver (see Figure 1). A detailed schematic of the electronic circuit is available on GitHub (https://github.com/AaronVelez/LI-6800_Lamp; see Data Availability Statement). The LED driver has three components. The first is a precision voltage‐to‐current converter/transmitter, the XTR110 chip (Table 1). The two other components are connected to the XTR110 chip, forming a feedback circuit that monitors and controls the current to the COB‐LED. A sense resistor monitors the current, and a P‐channel metal oxide semiconductor field‐effect transistor (P‐MOSFET) controls the current. While the current from an external 24‐V power supply flows through the sense resistor, a voltage drop across the resistor, proportional to the current, is measured by the XTR110 chip via the SOURCE‐SENSE pin. In turn, the XTR110 chip adjusts the voltage to the P‐MOSFET gate via the GATE_DRIVE_MOSFET pin. The sense resistor and the P‐MOSFET are both mounted on individual heatsinks. The regulated current flowing to the COB‐LED array is proportional to the input voltage on the LED_CTRL pin; this control voltage is set by the LI‐6800 console and has an operating range from 0 to 5 V.

#### Temperature control and monitoring

As the COB‐LED temperature increases, its light output decreases (CreeLED, 2021); therefore, the COB‐LED array is mounted on a standard CPU cooler, which consists of a heatsink and a four‐wire fan (Table 1). A precision negative temperature coefficient (NTC) thermistor measures the internal heatsink temperature. The thermistor signal (TEMP_SIG) is measured by both the LI‐6800 console and an Arduino‐compatible microcontroller board. The microcontroller board was a Trinket M0 board manufactured by Adafruit (Adafruit Industries, New York, New York, USA; https://www.adafruit.com/) (Table 1). The board's main processor is an ATSAMD21E18 32‐bit Cortex M0+ running at 48 MHz. It can be programmed in C/C++ or Python. In this project, Python was used (see the Software section). This board was selected because it is only 27 × 15.3 mm in size. The microcontroller monitors the heatsink temperature and controls the fan speed.

The microcontroller uses the measured temperature to adjust the fan speed according to a proportional‐integral‐derivative (PID) algorithm that calculates the speed needed to reach the setpoint. This regulation occurs via a 25‐kHz pulse‐width modulation (PWM) signal (FAN_PWM). The microcontroller measures the actual fan speed from the tachometer's digital signal on the FAN_TACH pin; then it encodes it into an analog signal and sends it to the LI‐6800 console via the FAN_SPEED signal. The heatsink temperature setpoint is defined by an analog signal from the LI‐6800 console (TEMP_CTRL). The LI‐6800 can generate analog signals from − 5 to 5 V; however, the microcontroller can only receive analog signals from 0 to 3.3 V. A Zener diode protects the microcontroller from potential damage by overvoltage by limiting the TEMP_CTRL voltage to 3.3 V; the Zener diode is depicted in the detailed electronic schematics at GitHub (see Data Availability Statement). The lamp design lacks hardware or software protection from improperly setting TEMP_CTRL voltage to values lower than 0 V; if this is done, the microcontroller could be permanently damaged.

In addition to the microcontroller, the LI‐6800 console measures the heatsink temperature by probing the same thermistor signal (TEMP_SIG). The LI‐6800 console uses the measured temperature to fine‐tune the voltage (LED_CTRL) needed to achieve the light intensity required by the user. The effect of the heatsink temperature on PPFD output was measured experimentally, and calibration coefficients were calculated using the collected data. Additional details are provided below.

#### Electronic protoboard, interface, and power supply

The XTR110 chip, microcontroller, a DC‐DC converter, and diverse discrete electronic components were assembled on a 45 × 45 mm protoboard with standard 0.1‐inch pitch (Appendix S1A). The sense resistor, P‐MOSFET, and COB‐LED generate heat that must be dissipated; therefore, they were placed on individual heatsinks. A shielded multiconductor cable with six individual conductor lines connects the lamp and the LI‐6800 console. At the LI‐6800 console side, a 25‐position D‐Sub standard connector attaches the individual signal lines to the *User I/O* port. At the lamp side, a printed circuit board (PCB) terminal block connects the individual conductor lines to the protoboard.

The lamp is powered by two independent sources. First, the COB‐LED driver and cooling fan are powered by an external 48‐W, 24‐V AC‐DC power supply. The COB‐LED driver is designed to use 24 V, but the fan needs 12 V. Therefore, a DC‐DC converter reduces the voltage from 24 to 12 V and delivers power to the fan. Ignoring inefficiencies, the COB‐LED driver and fan, at maximum power, consume at least 20.4 W. Second, the microcontroller is powered from the LI‐6800 console via the *User I/O* pin 14 (*Power5*). This line delivers 5 V when it is turned on from the LI‐6800 console.

#### Lamp housing and assembly

**Figure 2:**
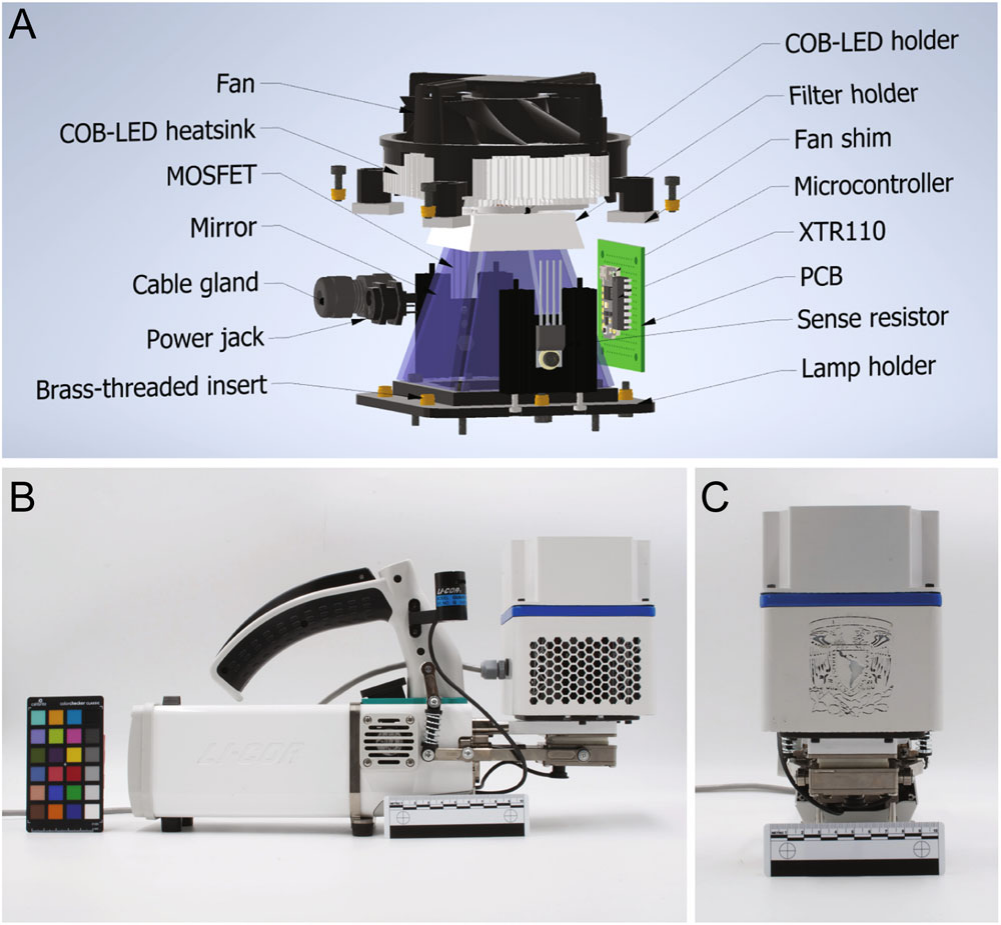
The mechanical design for a lamp for the LI‐6800 photosynthesis system. (A) The names of main internal parts in the 3D assembly. Notice that the main body and fan lid are not shown to depict internal parts. The complete 3D assembly is available in the GitHub repository (see Data Availability Statement). The lamp is shown attached to the LI‐6800 sensor head in side view (B) and front view (C).

A 3D‐printable housing was initially designed with SolidWorks (Dassault Systèmes, Vélizy‐Villacoublay, France; https://www.solidworks.com/) and later refined with Autodesk Inventor (Autodesk, San Francisco, California, USA; https://www.autodesk.com/). The lamp housing comprises three parts: the main body, fan lid, and lamp holder. The main body holds two heatsinks (which carry the P‐MOSFET and the sense resistor), the electronic PCB (carrying the microcontroller and LED driver), the CPU cooler assembly (carrying the COB‐LED and the thermistor), and four mirrors. An optional filter holder can be placed between the CPU cooler assembly and the main body (Appendix S1B). The filter holder accepts one‐inch‐diameter filters; in this paper, we characterized the lamp with and without using a diffuser filter (ED1‐S20, Table 1). The fan lid prevents the user from touching the fan blades and holds the CPU cooler assembly in place. The lamp holder secures the lamp housing to the LI‐6800 sensor head. The lamp holder is compatible with the Clear‐top Leaf Chamber (model 6800‐12A, LI‐COR Biosciences). Editable 3D models and ready‐to‐print files are available at GitHub, and the main internal components are depicted in Figure 2.

The lamp housing parts were printed with a stereolithography (SLA) 3D printer (model Form 2) using Black Resin v1.0 (Formlabs, Somerville, Massachusetts, USA). After 3D printing, parts were first cleaned with isopropyl alcohol using the Form Wash accessory equipment (Formlabs); then, parts were cured at 40°C for 60 min using the Form Cure accessory equipment (Formlabs). Later, the parts were painted with epoxy aerosol paint. Finally, heat‐set threaded brass inserts were fixed at the top and bottom of the main body to secure the fan lid and lamp holder using M3 screws. The lamp is shown installed on the LI‐6800 sensor head in Figure 2B and C, the model and manufacturer of all parts used are available in Table 1, and step‐by‐step fabrication instructions are provided in Appendix 1.

### Lamp and LI‐6800 software

#### Microcontroller code

The microcontroller is pre‐loaded with CircuitPython (version 8.2.2; https://github.com/adafruit/circuitpython), which is a beginner‐friendly, open‐source version of Python 3. In turn, CircuitPython is a derivative from MicroPython (version 1.20.0; https://micropython.org/). The microcontroller monitors and controls the COB‐LED heatsink and its fan using standard libraries, including *board*, *pulseio*, *time*, *math*, *digitalio*, and *analogio*. As CircuitPython does not have native PID algorithms, we programmed a simple PID controller using only basic functions. Our PID controller is based on published code (Kantor, 2020); the Python script used by the microcontroller in the lamp is available on GitHub (see Data Availability Statement).

The code loaded onto the microcontroller is a simple loop with five steps. The first step reads the analog signal at the TEMP_CTRL pin, which encodes the COB‐LED heatsink temperature setpoint defined by the LI‐6800 console. Step two measures the actual COB‐LED temperature. This is done by measuring the voltage at the TEMP_SIG pin and calculating the thermistor resistance using Ohm's law and the known thermistor excitation voltage (3.3 V). Then, thermistor resistance is converted to thermistor temperature using the manufacturer's temperature‐resistance equations and coefficients. In step three, the fan speed is read at the FAN_TACH pin. The microcontroller then encodes the fan speed into an analog signal and sends it to the LI‐6800 console at the FAN_SPEED pin. During step four, the PID algorithm is computed; it calculates the PWM duty cycle needed to reach the COB‐LED heatsink temperature setpoint. Finally, during step five, the calculated PWM duty cycle is output to the FAN_PWM pin.

#### LI‐6800 custom configuration and background program

**Figure 3:**
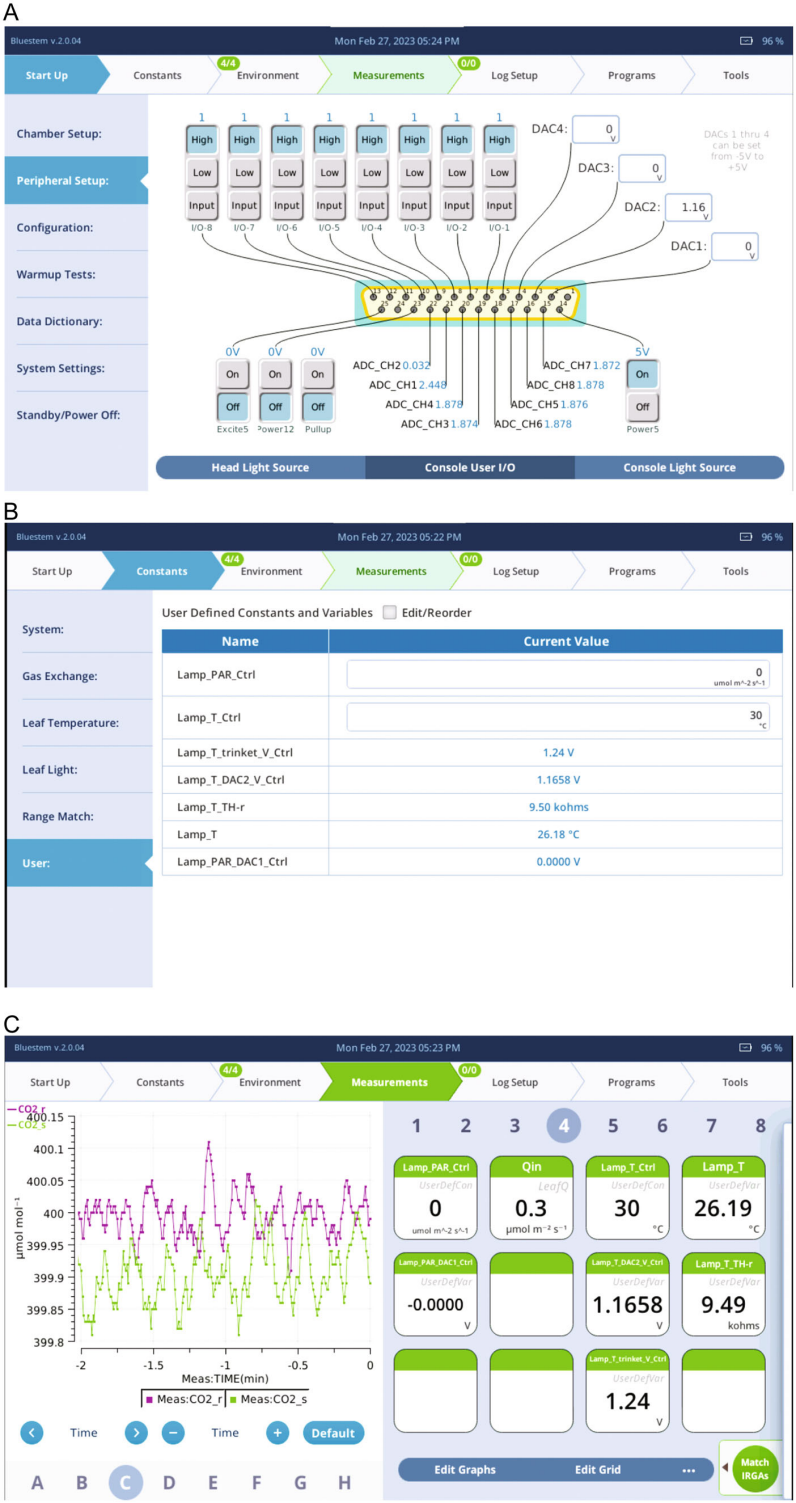
The LI‐6800 display screens while using the lamp. (A) The custom *User I/O* state loaded from the configuration file. Note that the 5 V power must be ON in order to power the microcontroller. (B) The user constants and variables added by the configuration file. Note that the user constants *Lamp_PAR_Ctrl* and *Lamp_T_Ctrl* are settable fields to aid lamp use. The non‐settable fields are user variables and perform all necessary calculations to control the lamp programmatically. (C) The configuration file also adds user constants and variables to the grid display on the Measurements tab.

The lamp's COB‐LED driver and microcontroller obtain the PPFD and temperature setpoints from the LI‐6800 console's *User I/O* channels DAC1 and DAC2, respectively. DAC1 is connected to the LED_CTRL pin and DAC2 to the TEMP_CTRL pin (see *User I/O* D‐Sub connector pin mapping in the LI‐6800 manual [LI‐COR Biosciences, 2021]). Although the lamp can be controlled by changing DAC1 and DAC2 voltage (Figure 3A), changing these values manually is impractical because the mapping between the DACs voltages and PPFD or temperature setpoints depends on the calibration coefficients. To simplify the lamp control by the user, therefore, we developed a custom LI‐6800 configuration file and programmed an LI‐6800 BP named *PAR_Ctrl.py*. The custom configuration file and BP can be downloaded from GitHub.

The custom LI‐6800 configuration file has four configuration fields: user definitions (*UsrDef*), stability definitions (*Stability*), configuration of variables at the *Measurements* tab (*Grids*), and the state of the *User I/O* channels (*Aux*). Note that *User I/O* was formerly known as *Auxiliary I/O*; in the LI‐6800 Bluestem OS v2.0 manual, the term *Auxiliary I/O* is still used (LI‐COR Biosciences, 2021). In this study, we used the Bluestem OS v2.1.11, which uses the new term *User I/O*.

When the *UsrDef* field is loaded from the configuration file, user constants and variables (*UsrDef*) are added. User‐defined constants are shown at the LI‐6800 Console *Constant > User* tab as user‐settable fields (Figure 3B). The user, therefore, can input the desired COB‐LED PPFD and temperature setpoints in the target units; those are μmol∙m^−2^∙s^−1^ and °C for the PPFD and temperature, respectively. In turn, the loaded user variables calculate the DAC1 and DAC2 voltages that are required to reach the desired setpoints. The equations and calibration coefficients needed to calculate these conversions are within the configuration file (*UsrDef* field). Notice that the user variables only calculate the required DAC1 and DAC2 voltages, but they do not set those voltage values in the LI‐6800 *Console User I/O* tab. Nonetheless, the *PAR_Ctrl.py* BP automatically sets DAC1 voltage to the required voltage. The *PAR_Ctrl.py* BP needs to be run by a user or by another BP. Once started, *PAR_Ctrl.py* BP runs in the background, allowing the user to control the lamp with ease. As the user variables are also settable by any BP, the lamp can be fully controlled programmatically.

When the *Aux* field is loaded from the configuration file, the 5‐V power supply from the *User I/O* is turned on (pin 14 in the D‐Sub connector) and powers the microcontroller. The *Aux* field also sets the DAC2 voltage to 1.1658 V, which corresponds to a COB‐LED heatsink temperature setpoint of 30°C; this precludes the possibility of the user forgetting to turn on the microcontroller and setting the heatsink temperature. The LI‐6800 *User I/O* channel ADC_CH1 measures the voltage at the TEMP_SIG pin. Using the read voltage and calibration coefficients, a user variable calculates the COB‐LED heatsink temperature. Similarly, the ADC_CH2 channel measures voltage at the TEMP_SIG pin, which is used to calculate fan speed. When the *Grid* field is loaded from the configuration file, the variables associated with lamp operation are displayed at the LI‐6800 console *Measurements* tab (Figure 3C) for convenient monitoring during measurements.

### Lamp calibration and characterization

#### Lamp‐emitted light characterization and calibration

**Figure 4:**
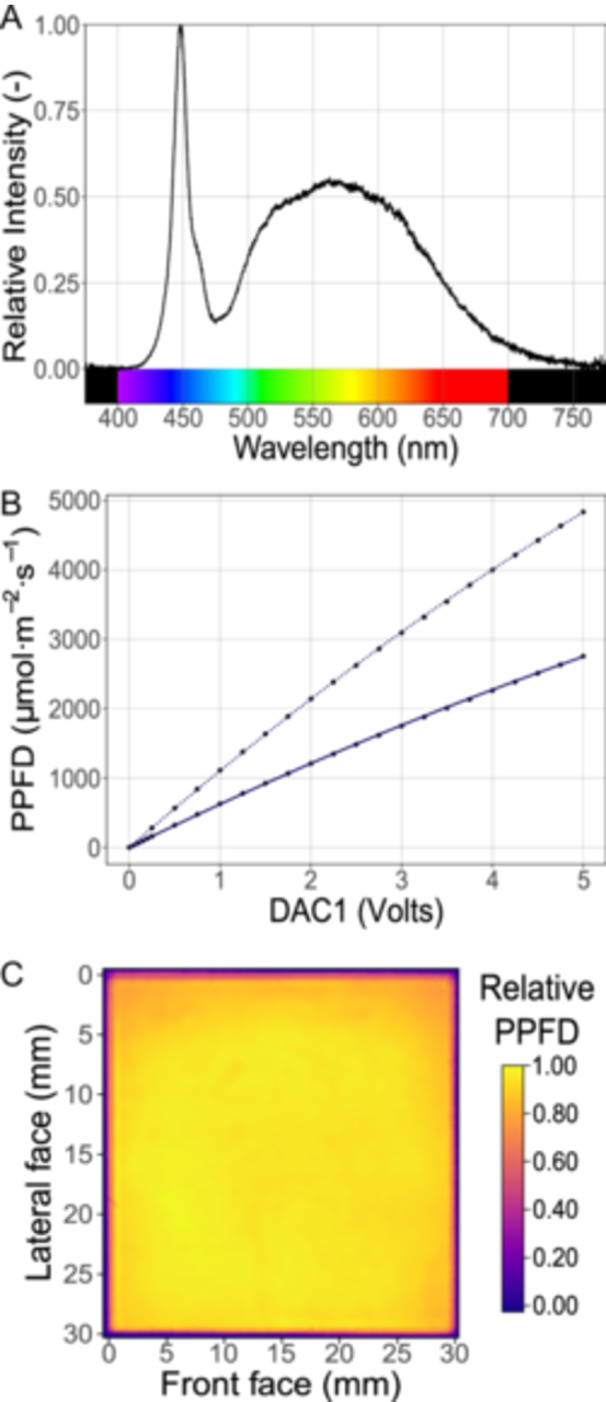
Characterization of the lamp for the LI‐6800 photosynthesis system. (A) The spectral distribution of light at the leaf plane with the lamp set to 1% of its power and with an integration time of 1 ms. (B) The resulting photosynthetically active photon flux density (PPFD) at the leaf plane, controlled by DAC1 voltage. The dotted line represents the performance of the lamp without a diffuser, while the solid line represents the performance with the ED1‐S20 diffuser. (C) The relative PPFD distribution at the leaf plane using the 3 × 3 cm aperture insert and the ED1‐S20 diffuser. The PPFD distribution was measured over a 150 × 150 grid, yielding a total of 22,500 measurements, each taken at 0.2‐mm intervals; a margin of 0.4 mm is included. This image has been cosine corrected. For further details, refer to the “Lamp calibration and characterization” subsection.

We measured the spectral distribution of the emitted light using an ultraviolet–visible spectrophotometer (AvaSpec‐ULSi‐series; Avantes, Apeldoorn, The Netherlands). The spectrophotometer measurements were corrected through a transfer function, which was derived by comparing a measured spectrum of the sun on a clear day to the American Society for Testing and Materials (ASTM) reference air mass 1.5 global tilt spectrum, part of the G‐173 spectra (American Society for Testing and Materials, 2024). Measurements were conducted with the COB‐LED installed into the lamp housing, both with and without the ED1‐S20 diffuser and Propafilm C (LI‐COR Biosciences) leaf chamber covering film. The ED1‐S20 and Propafilm C are achromatic between 400 and 750 nm (Appendix S2), indicating they do not alter the shape of the COB‐LED spectrum. The spectral measurements were performed with the lamp set to 1% of its power and with an integration time of 1 ms (Figure 4A). The light emitted in the photosynthetically active range (400–700 nm) constitutes 97.6% of the total emitted light. The COB‐LED exhibits a red to far‐red ratio of 7.43, and the calculated phytochrome photostationary state (Sager et al., 1988) is 0.845. We cannot directly compare these values to commercially available lamps (like the LI‐6800‐02 and ‐03) because the manufacturers do not disclose these specifications and do not publish the spectral measurements.

The custom driver circuit increases the current to the COB‐LED in response to the voltage at the LED‐CTRL pin, which is connected to the LI‐6800 DAC1 *User I/O* channel. In turn, the light emitted by the COB‐LED is proportional to the current; however, as with any LED, there are also small effects of the temperature on the emitted light intensity. As the COB‐LED temperature increases, the emitted light, at any given current, slightly decreases. The effect of the leaf chamber aperture inserts also needs to be considered. The leaf chamber (model 6800‐12A, LI‐COR Biosciences) has three aperture inserts (3 × 3, 2 × 3, and 1 × 3 cm). When using an insert with a smaller aperture, more metal surface reflects the emitted light back to the mirrors, and then back to the leaf, resulting in a slight increase in PPFD at the leaf surface. To accurately control the COB‐LED, therefore, we performed a calibration that considers these three variables defining PPFD at the leaf surface. The detailed calibration procedure is outlined in Appendix 2. In summary, we recorded the COB‐LED heatsink temperature and the actual PPFD inside the leaf chamber using a light meter (model LI‐250A, LI‐COR Biosciences) equipped with a quantum sensor (LI‐190R Quantum, LI‐COR Biosciences) at DAC1 voltages from 0 to 5 V. This procedure was repeated twice with each of the three aperture inserts. As expected, PPFD increases in response to DAC1 voltage (Figure 4B). Using SAS Studio (version 3.81; SAS Institute, Cary, North Carolina, USA), we evaluated several multiple linear regression models using DAC1, COB‐LED heatsink temperature, and aperture insert area as predictor variables. The selected model is as follows:

(1) $${\mathrm{PPFD}}_{\mathrm{leaf}}=a\;\cdot \;\mathrm{DAC}1+{b\;\cdot \;\mathrm{DAC}1}^{2}+c\;\cdot \;\mathrm{DAC}1\;\cdot \;\mathrm{Lam}p_{\mathrm{Temp}}+d\;\cdot \;\mathrm{DAC}1\;\cdot \;\mathrm{Insert}\_\mathrm{Area}$$

where *PPFD*_*leaf*_ is the predicted PPFD at the leaf surface within the leaf chamber in μmol∙m^−2^∙s^−1^, *DAC1* is the voltage at the LI‐6800 *User I/O* DAC1 channel in volts, *Lamp*_*Temp*_ is the COB‐LED heatsink temperature in °C, *Insert_Area* is the aperture insert area in cm^2^ (the 3 × 3 aperture inserts result in an *Insert_Area* of 9 cm^2^), and the letters *a*, *b*, *c*, and *d* represent regression coefficients. The estimated coefficients are provided in Table 2; the resulting *r* ^2^ for the selected model and the used calibration data is 0.999. This means that the calibration equation can accurately predict the resulting PPFD at the leaf surface. As the calibration equation and coefficients are included in user variables within a LI‐6800 custom configuration file, the user can control the PPFD of any experiment by running the *PAR_Ctrl.py* BP script and adjusting, manually or programmatically, a user constant.

**Table 2:** Calibration coefficients.

Calibration	Estimated coefficients			
	a	b	c	d
PPFD at leaf surface without diffuser (Eq. 1)	1198.290537	−31.758607	−1.948934	−2.515517
PPFD at leaf surface with ED1‐S20 diffuser (Eq. 1)	674.391795	−16.509904	−0.725546	−1.463820
Required DAC2 voltage (Eq. 3)	12199	147438769	45440000	2272

In addition to the calibration defining the relation between DAC1 control voltage and PPFD at leaf level, we developed a way for the user to measure PPFD during an experiment by using the photodiode sensor built into the leaf chamber. According to the LI‐6800 manual (LI‐COR Biosciences, 2021), the built‐in photodiode sensor reading is used to calculate the variable *Q*_*amb_in*_, as follows:

(2) $$Q_{\mathrm{amb}\_\mathrm{in}}=\frac{S_{qin}\;\cdot \;\left(2500000\right)\;\cdot \;I_{qin}}{2\;\cdot \;16\;\cdot \;65536\;\cdot \;200}$$

where *Q*_*amb_in*_ is the ambient light PPFD inside the leaf chamber in μmol∙m^−2^∙s^−1^, and *I*_*qin*_ is the photodiode raw counts from the ADC converter; the calibration factor *S*_*qin*_ is stored within the leaf chamber memory as a multiplier, with units μmol∙m^−2^∙s^−1^∙μA^−1^. Without recalibration of the *S*_*qin*_ multiplier, *Q*_*amb_in*_ overestimates the COB‐LED PPFD output (Appendix S3). This is because the built‐in photodiode is fine‐tuned for solar irradiance and is placed closer to the COB‐LED than the external quantum sensor, which was placed at the leaf plane. Further insights on this effect are provided in the LI‐6800 manual (LI‐COR Biosciences, 2021; see the *Measuring ambient light* and *Transmittance factors* subsections in chapter 3). Up to a PPFD of around 2700 μmol∙m^−2^∙s^−1^, however, there is a perfect linear correlation between *Q*_*amb_in*_ and the measured PPFD using an external quantum sensor, resulting in an *r* ^2^ = 0.9997. Above 2700 μmol∙m^−2^∙s^−1^, the *Q*_*amb_in*_ signal saturates; therefore, the built‐in photodiode can be used to monitor PPFD up to 2700 μmol∙m^−2^∙s^−1^ if recalibrated with a new *S*_*qin*_ value. The new *S*_*qin*_ value was calculated by dividing the original *S*_*qin*_ by the slope of the linear relation between *Q*_*amb_in*_ and the measured PPFD using the external quantum sensor. The estimated *S*_*qin*_ is 6.87492 μmol∙m^−2^∙s^−1^∙μA^−1^. The new multiplier results in a perfect linear relation between *Q*_*amb_in*_ and the PPFD value measured with an external quantum sensor (Appendix S3).

Once the built‐in photodiode is recalibrated, *Q*_*amb_in*_ can be used to feedback PPFD control by Eq. 1. Although, after a calibration, Eq. 1 is enough to control PPFD, further DAC1 voltage fine adjustment by *Q*_*amb_in*_ feedback can compensate for the decrease in COB‐LED intensity associated with hours of use. However, such a decrease is slow. According to manufacturer tests, the used COB‐LED retains 90% of its irradiance output even after 66,500 h of use at maximum power (CreeLED, 2023). Additionally, not all leaf chambers are equipped with an internal photodiode. Therefore, we developed two ways to control PPFD, which are with and without *Q*_*amb_in*_ feedback. Both of these control approaches have advantages and disadvantages. The approach not using feedback allows the lamp to be used on any chamber and at any intensity, but it requires recalibration as the COB‐LED gets older. Using the feedback approach requires less frequent recalibration as the COB‐LED gets older; however, it will not work with all leaf chambers, and it only works at a PPFD lower than 2700 μmol∙m^−2^∙s^−1^.

Because the lamp housing holds four mirrors and positions the COB‐LED either 82.1 or 84.965 mm above the leaf plane (depending on whether the ED1‐S20 diffuser is installed), the emitted light is expected to uniformly illuminate the leaf. To test this, we used a CNC machine (model 3018‐PROVer V2; SainSmart, Lenexa, Kansas, USA) to sample irradiance every 0.2 mm. The sampling probe was a bare fiber optic with a 200‐μm core (model M38L01; Thorlabs, Newton, New Jersey, USA), attached to the CNC machine via an upgraded aluminum spindle holder. Irradiance was measured using an amplified photodiode (PDA100A2, Thorlabs) with a 700‐nm short‐pass filter (model 84‐701; Edmund Optics, Barrington, New Jersey, USA). The photodiode signal was digitized with a multifunctional DAQ (model PCIe‐6361; National Instruments, Austin, Texas, USA). The coolant on signal was used to synchronize the CNC with the DAQ via the GRBL v1.1 G‐code. A custom Python script generated the G‐code instructions for the XY scans, and a custom LabVIEW 2018 (National Instruments) virtual instrument (VI) collected and processed the photodiode signal measurements. As the CNC machine induced some noise in the synchronization and photodiode signals, resistor‐capacitor low‐pass filters were used to clean these signals. To further reduce noise in the photodiode signal, 2500 voltage measurements were taken per XY point, and a finite impulse response filter was applied before averaging these samples to produce a single measurement for each XY point.

The maximum angle between the COB‐LED normal plane and the fiber optic was 14.48°, and it was achieved when measuring the corner of the 3 × 3 cm insert, with no diffuser installed, as the COB‐LED is closer to the leaf plane when no diffuser is used. Because the used fiber optic has a numerical aperture of 0.39, its maximum acceptance angle is 22.95°, which is larger than the maximum of 14.48° achieved during measurements; nevertheless, a correction for the angle of incidence is needed. Lambert's cosine law describes the relationship between irradiance perpendicular to a light source (denoted as *I*_*zero*_) and irradiance at an angle *θ* deviating from the normal (denoted as *I*_*angle*_). This relationship is expressed as:

(3) $$I_{\mathrm{angle}}=I_{\mathrm{zero}}\;\cdot \;cos(\theta )$$

To solve for *I*_*zero*_ and normalize it to one, we rearrange the equation:

(4) $$I_{\mathrm{zero}}=\frac{1}{cos(\theta )}$$

This normalization, referred to as the cosine correction (Appendix S4), is applied only to the fraction of light that reached the fiber optics probe directly. This direct component is the light that reaches the probe without reflection from any mirrors:

(5) $${\mathrm{PD}}_{\mathrm{corrected}}=\left({\mathrm{PD}}_{\mathrm{direct}}\;\cdot \;\frac{1}{\cos(\theta )}\right)+{\mathrm{PD}}_{\mathrm{indirect}}$$

where PD stands for photodiode measurements in mV and *θ* is the angle of incidence in radians. *PD*_*direct*_ and *PD*_*indirect*_ are defined as follows:

(6) $${\mathrm{PD}}_{\mathrm{direct}}={\mathrm{PD}}_{\mathrm{raw}\_\mathrm{black}}$$

(7) $${\mathrm{PD}}_{\mathrm{indirect}}={\mathrm{PD}}_{\mathrm{raw}\_\mathrm{mirror}}-{\mathrm{PD}}_{\mathrm{raw}\_\mathrm{black}}$$

where *PD*_*raw_mirror*_ is the photodiode measurements taken with the lamp assembled as described in Appendix 1, and *PD*_*raw_black*_ is the photodiode measurements taken with the lamp mirrors covered with a plastic sheet air‐brushed with a black paint with a reflectance of only 0.06, according to manufacturer specifications (Musuo Black; Koyo Orient Co., Saitama, Japan).

Because aligning the lamp to the CNC *x*‐ and *y*‐axes with a precision of 0.2 mm or better was not practical, we added a 3‐mm margin to our scans. Using the *OpenCV* library for Python, we identified the aperture in the raw measurements by applying Otsu's automatic threshold algorithm. We then calculated the centroid of the largest contour in the resulting binary image and used it as a pivot point to rotate the image for proper alignment with the image's edge. A rectangle matching the expected aperture size was drawn, centered on the calculated centroid. This approach allowed us to segment the pixels corresponding to the aperture and align them with their corresponding pixels from two independent measurements: mirror and black measurements (refer to Eq. 7). Finally, we applied the cosine corrections as described in Eq. 5. All LabVIEW VIs and Python scripts are available in our GitHub repository.

The raw and corrected homogeneity measurements for all inserts and both lamp versions are available in Appendices S5 and S6, and a summary of all homogeneity measurements is provided in Appendix S7. Figure 4C shows the normalized irradiance homogeneity for the lamp using the ED1‐S20 diffuser and the 3 × 3 cm insert. Both versions presented a coefficient of variation (CV) lower than 10% in at least 97% of the area, regardless of the insert used. Compared with commercially available lamps (LI‐6800‐02 and ‐03), the lamp presented here has more area with a CV lower than 10%. The use of the ED1‐S20 diffuser improves the homogeneity, but the effect is only around 1%. Considering its high cost, we suggest that its use is optional.

#### Lamp temperature control characterization and calibration

The COB‐LED heatsink temperature setpoint is set by the voltage at the TEMP_CTRL pin, which is connected to the LI‐6800 DAC2 *User I/O* channel. As stated in the hardware section, however, a Zener diode reduces the voltage generated by the LI‐6800 to protect the microcontroller circuits. Using a multimeter (model 87V; Fluke Corporation, Everett, Washington, USA), the voltage reduction by the Zener diode was measured (Appendix S8). To define the DAC2 voltage required to inform the microcontroller temperature setpoint, these measurements were fitted to the following equation using the *curve_fit* function of the *scipy* Python library.

(8) $${\mathrm{DAC}2}_{r}=\frac{a-\sqrt{b-c\;\cdot \;{\mathrm{TEMP}\_\mathrm{CTRL}}_{t}}}{d}$$

where *DAC2*_*r*_ is the DAC2 voltage required to reach the target voltage at the microcontroller side (*TEMP_CTRL*_*t*_), and the letters *a*, *b*, *c*, and *d* represent the regression coefficients. Step‐by‐step instructions to calculate the regression coefficients are outlined in Appendix 3, and the estimated coefficients are provided in Table 2. In turn, *TEMP_CTRL*_*t*_ is defined by the following equation:

(9) $$\begin{array}{ccl} {TEMP\_CTRL}_{t} & = & (Setpoint-Min_{Temp})\cdot \;\frac{3.3}{Max_{Temp}-Min_{Temp}} \end{array}$$

where *Setpoint* is the user‐defined COB‐LED heatsink temperature setpoint. The variables *Min*_*Temp*_ and *Max*_*Temp*_ are the minimum and maximum selectable temperature setpoints, respectively. Equations 8 and 9 are programmed within the LI‐6800 custom configuration file, while the microcontroller software only needs Eq. 9. Within the microcontroller software and custom LI‐6800 configuration file, *Min*_*Temp*_ and *Max*_*Temp*_ are set at 15°C and 55°C, respectively.

Although the lamp software allows the user to set a heatsink temperature setpoint between 15°C and 55°C, those temperatures are not always reachable. To characterize the ability of the fan and heatsink to reach the setpoint temperature, we varied the COB‐LED intensity from 0% to 100% and changed the heatsink temperature setpoint from 2°C lower up to 14°C higher than room temperature. However, we did not try all combinations of COB‐LED intensity and temperature setpoint. For example, at high COB‐LED intensity, it is not feasible to use a setpoint close to room temperature; hence, we selected 884 combinations that were closest to the attainable range. The results are shown in Appendix S9. At maximum COB‐LED intensities, the fan does not cool the heatsink to a temperature less than 10°C above room temperature; however, even if the heatsink heats up to 80°C, the attainable PPFD is more than 4250 or 2500 μmol∙m^−2^∙s^−1^, depending on whether the ED1‐S20 diffuser is installed or not (Appendix S10). This PPFD is as high as commercially available alternatives and is more than enough to perform most plant science experiments, which require a PPFD of less than 2000 μmol∙m^−2^∙s^−1^ (e.g., an A‐PPFD curve; Coe and Lin, 2018). The lamp's technical specifications are compared with those of commercially available lamps in Appendix S11.

### Lamp example usage

To demonstrate the use of the lamp, we performed A‐PPFD curves using bean (*Phaseolus vulgaris* L.) plants. Bean seeds, cv. Pinto Saltillo, were purchased from Bidasem (Celaya, Guanajuato, Mexico) and were sown directly on fresh soil mix on 16 February 2024. Each batch of soil mix contained 250 L of peat moss (REMIX 1; Rėkyva Joint‐Stock Company, Šiauliai, Lithuania), 200 L of perlite (Multiperl Hortícola; Grupo Perlita, Gómez Palacio, Durango, Mexico), 100 L of vermiculite (Disa Sustratos Agrícolas, Mexico City, Mexico), 100 L of coconut fiber (Hydro Environment, Tlalnepantla, Mexico), 60 L of silica sand (Decoproductos, Mexico City, Mexico), 2 kg of dolomite limestone (Zeolitech, Cuernavaca, Morelos, Mexico), 2 kg of slow‐release N‐P‐K (16‐16‐16) fertilizer (Yara Mila UNIK16; Yara, Oslo, Norway), 875 g of chelated micronutrient mix (Kelatex Multi; Cosmocel, Monterrey, Nuevo León, Mexico), 375 g of magnesium sulphate heptahydrate (Industrias Peñoles, Mexico City, Mexico), and 150 g of calcium sulphate (Grow Depot Mexico, Torreón, Coahuila, Mexico). Plants were grown in a glass greenhouse located within the University facilities in the city of León, Guanajuato, Mexico. When plants were five weeks old, the youngest, fully expanded leaf was measured.

**Figure 5:**
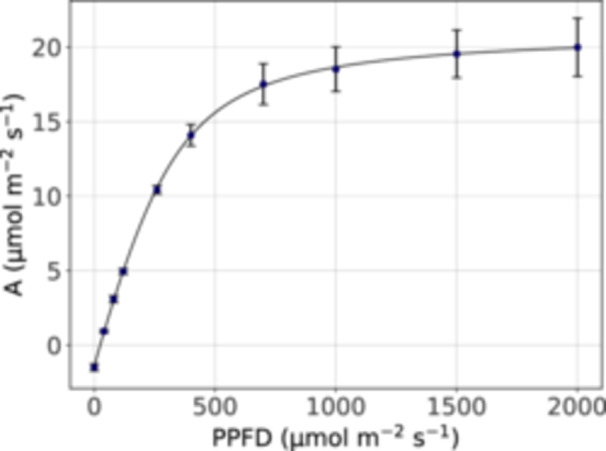
An A‐PPFD curve measured using the lamp developed here and the LI‐6800 photosynthesis measuring system. Each point is the mean of five biological replicates. Error bars show the standard deviation of the mean. Using the model depicted in Eq. 10, the regression line is calculated using the parameter mean estimate shown in Table 3.

Using the lamp presented here, A‐PPFD curves were performed with the LI‐6800 photosynthesis measuring system. A Python script was used to perform the measurements automatically; the script is available in the GitHub repository, and detailed instructions for usage are provided in Appendix 4. The leaf temperature was controlled at 25°C. To avoid ΔCO_2_ values higher than 30 μmol∙m^−2^∙s^−1^, the flow rate was set between 275 and 775 μmol∙m^−2^∙s^−1^. The fan speed was set at 9000 rpm, the CO_2_ concentration at the sample infrared gas analyzer (IRGA) was set at 400 ppm, and the vapor pressure deficit (VPD) at 1.2 kPa. The assimilation rate was measured at 10 PPFD levels (0, 40, 80, 120, 260, 400, 700, 1000, 1500, and 2000 μmol∙m^−2^∙s^−1^); after each change in PPFD, the script paused to allow levels to stabilize before logging. The reference and sample IRGAs were matched before each log. The raw data are available at GitHub and follow the reporting format defined for leaf‐level gas exchange data (Ely et al., 2021). The resulting A‐PPFD curves are shown in Figure 5. The photosynthetic parameters were estimated using a non‐rectangular hyperbola model (Busch et al., 2024) according to the following equation:

(10) $$A=\frac{\Phi_{CO_{2}}\;\cdot \;PPFD+(A_{sat}+R_{d})-\sqrt{\Phi_{{\mathrm{CO}}_{2}}\;\cdot \;\mathrm{PPFD}+{\left(A_{\mathrm{sat}}+R_{d}\right)}^{2}-4\;\cdot \;\theta \;\cdot \;\Phi_{{\mathrm{CO}}_{2}}\;\cdot \;\mathrm{PPFD}\;\cdot \;(A_{\mathrm{sat}}+R_{d})}}{2\theta }-R_{d}$$

where *A* and *A*_*sat*_ are the CO_2_ assimilation rate and maximum assimilation rate, respectively; *PPFD* is the incident irradiance; the parameter *ΦCO*_*2*_ is the maximum quantum efficiency for CO_2_ assimilation; and *R*_*d*_ is the rate of respiratory CO_2_ release from mitochondria. All the parameters were estimated using Microsoft Excel by minimizing the least squares error. The photosynthetic parameter estimates (Table 3) are intrinsically linked to the plant's specific physiology and can be influenced by the spectral distribution of the measuring lamp (Domurath et al., 2012; Lanoue et al., 2017; Liu and van Iersel, 2021). In general, a light source that emits multiple wavelengths tends to result in better plant performance. In this study, therefore, the use of white measuring light instead of monochromatic LEDs is potentially advantageous considering the results obtained by others. For example, Lanoue et al. (2017) found that using a white measuring light led to a higher maximum leaf net carbon exchange rate (similar to *A*_*sat*_) than monochromatic red, green, or blue light. Likewise, Liu and van Iersel (2021) observed a higher maximum gross assimilation rate and ΦCO_2_ when A‐PPFD curves were measured with a combination of red, green, and blue light compared to blue light alone. These effects can be attributed to the different absorbance of light colors by the leaf and the associated varying penetration depths within leaf tissue (Liu and van Iersel, 2021), as well as their differential impacts on stomatal opening (Lanoue et al., 2017). Nonetheless, other effects such as RUBISCO activation, chloroplast movements, and state transition induction cannot be discarded.

**Table 3:** Photosynthetic parameters.

**Parameter**	**Mean**	**SD**
*ΦCO*_*2*_	0.0576	0.0028
*A*_*mat*_	21.03	2.09
*R*_*d*_	1.38	0.13
*θ*	24.36	2.66

## CONCLUSIONS

Here, we report the design, construction, testing, and use of a lamp compatible with the LI‐6800 photosynthesis system. The lamp can be built using a basic electronics lab and a 3D printer. We share calibration instructions, which only require equipment commonly available in plant science laboratories. The use of the ED1‐S20 diffuser results in a marginal improvement in homogeneity; therefore, we suggest its use only if the extra cost is justified by experiment requirements. The lamp is a cost‐effective alternative to perform photosynthesis research coupled with the popular LI‐6800 photosynthesis measuring system.

Despite its advantages, there are limitations to consider. The lamp currently lacks robust protection against improper voltage settings in control lines, which could lead to lamp damage. Additionally, the absence of an integrated photodiode for feedback control means that light intensity adjustments depend on the chamber photodiode and/or frequent calibrations. Although we developed BP and an LI‐6800 custom configuration file to allow programmatic control over the lamp, its use is not integrated into the LI‐6800 native lamp controls. Furthermore, unlike commercial alternatives such as the LI‐6800‐03, the measuring spectrum cannot be adjusted. Future improvements should address these limitations by including reverse voltage protection, a dedicated photodiode, and independently dimmable LEDs of varying spectra. Additionally, using the LI‐6800 MQTT communication system would allow for better native software integration.

## AUTHOR CONTRIBUTIONS

A.I.V.R., J.d.D.M., and H.C.A. contributed to the hardware design. A.I.V.R., J.d.D.M., and U.G.P.G. programmed the required software. A.I.V.R., J.d.D.M., U.G.P.G., and A.M.J. performed the lamp characterization and calibration. A.I.V.R., J.d.D.M., and U.G.P.G. participated in the biological measurements. A.I.V.R., U.G.P.G., and J.V.M. supervised BSc students working on the project. A.I.V.R., J.V.M., and I.H.M. conceptualized the idea. A.I.V.R., J.V.M., and I.H.M. drafted the paper. A.I.V.R. and I.H.M. secured the funding. All authors reviewed and approved the final version of the manuscript.

### Open Research Badges

This article has earned Open Data and Open Materials badges. Data and materials are available at https://github.com/AaronVelez/LI-6800_Lamp.

## Supporting information


**Appendix S1.** Mechanical details of a lamp for the LI‐6800 photosynthesis system.
**Appendix S2.** Spectral effects of the ED1‐S20 diffuser (A) and Propafilm C leaf chamber covering film (B).
**Appendix S3.** Correlation between irradiance measured by an external quantum sensor and the internal photodiode.
**Appendix S4.** Cosine corrections for the lamp without (A) and with the ED1‐S20 diffuser installed (B).
**Appendix S5.** Photosynthetic photon flux density (PPFD) distributions at the leaf plane, using the 3 × 3 cm, 2 × 3 cm, and 1 × 3 cm aperture inserts, without the diffuser installed.
**Appendix S6.** Photosynthetic photon flux density (PPFD) distributions at the leaf plane, using the 3 × 3 cm, 2 × 3 cm, and 1 × 3 cm aperture inserts, with the ED1‐S20 diffuser installed.
**Appendix S7.** Table summarizing the lamp photosynthetic photon flux density (PPFD) uniformity scans.
**Appendix S8.** Effect of the Zenner diode protection.
**Appendix S9.** Attainable COB‐LED heatsink temperature setpoint.
**Appendix S10.** Attainable photosynthetic photon flux density (PPFD) without the diffuser (A) and using the ED1‐S20 diffuser (B).
**Appendix S11.** Technical specifications for the lamp described in this paper, alongside two commercially available lamps from LI‐COR Biosciences (Lincoln, Nebraska, USA).

## Data Availability

The data and metadata from the A‐PPFD measurements, along with all hardware and software to fabricate the open‐source LED lamp, are available at the GitHub repository (https://github.com/AaronVelez/LI-6800_Lamp). The hardware is released under a CERN Open Hardware License Version 2, weakly reciprocal variant; the software is released under a GNU Lesser General Public License v3.0.

## Appendix 1 Step‐by‐step instructions to build a lamp for the LI‐6800 photosynthesis measuring system.

Note: All required parts and materials are listed in Table 1. The CAD files and electronic schematics are available at GitHub (https://github.com/AaronVelez/LI-6800_Lamp). Printing instructions using the STL files are provided here; however, the original files in IPT and STP formats are also provided in case the user needs to modify the CAD design. The provided instructions apply to the Form 2 3D printer from FormLabs; however, the protocol can be adjusted for use with other 3D printers.

### 3D printing of the lamp housing

1. From the GitHub repository, download all files in the *Ready‐to‐Print STL files* directory.
2. Download and install the PreForm software from https://formlabs.com/software/.
3. Open the *Main Body.stl* file with PreForm.
4. Orient the part so its base sits on top of the printing platform.
5. Generate 3D printing supports using default settings.
6. At the *Job Setup* menu, select printer (Form 2), material (Black resin), and the *z*‐axis resolution (50 μm).
7. Print the job.
8. Remove the printing platform from the printer and wash the resin residues with isopropyl alcohol. If available, use the *Form Wash* equipment (FormLabs); otherwise, do it manually.
9. Remove the part from the printing platform. Do not remove the 3D printing supports yet.
10. Cure the part using the *Form Cure* equipment (FormLabs). Set the curing temperature and time to 40°C and 60 min, respectively. Make sure to place the part inside the equipment before it warms up to prevent deformations. Curing at 60°C, as recommended by the manufacturer, tends to deform big parts.
11. Remove the 3D printing supports using pliers.
12. Using wet sandpaper, smooth the part surfaces. Start with coarse sandpaper and gradually use smoother sandpaper; for example, use sandpaper with grit numbers 120, 320, and 600, in that order.
13. Paint the exterior of the part with epoxy paint. Do not paint the interior. Apply fine layers, allowing each layer to dry before applying the next one. Three layers are recommended.
14. Repeat steps 3 to 13 with the *Fan Lid.stl* and *Spacer.stl* parts.
15. Repeat steps 3 to 12 with the *Fan shim.stl*, *Filter Holder_25.4 mm.stl*, *Lamp Holder.stl*, and *Retaining ring.stl* parts. Do not paint these parts as it would prevent proper fit.

### Adding threads

The following instructions assume that the exact threaded brass inserts and heatsinks are used as indicated in Table 1. Adjust the drilling and tapping bits if alternative threaded inserts and/or heatsinks are used.

1. From the GitHub repository, download the *Lamp.iam* assembly file as well as files for all individual parts (i.e., all **.ipt* files).
2. Visualize the lamp assembly using the AutoDesk Viewer online tool (https://viewer.autodesk.com/designviews).
3. By exploring the assembly with the online viewer, identify the places on the main body of the lamp where brass‐threaded inserts need to be installed. The spots are marked with 3‐mm holes, and there are four places on top and six at the bottom.
4. At the identified spots, drill a 4‐mm‐depth hole using a number 8 drill bit (5.0546 mm in diameter).
5. Install the threaded inserts by pressing them into place with a hot soldering tip. If the brass inserts fall after installation, use a drop of superglue to secure them again to the lamp main body.
6. By exploring the assembly with the online viewer, identify the places of the TO‐220 heatsinks where threads need to be tapped.
7. At the identified spots, machine an internal thread using a M2.5 tapping bit.
8. Refer to the LED holder datasheet (https://tools.molex.com/pdm_docs/sd/1805550002_sd.pdf) to identify the places of the CPU cooler heatsink where threads need to be tapped.
9. At the identified spots, drill with the adequate drill bit before machining an internal thread using a M2.5 tapping bit.

### Protoboarding electronic components

1. From the GitHub repository, download the *Lamp_schematics.pdf* file, which contains detailed electronic schematics.
2. Download the Supporting information and refer to Appendix S1.
3. Using the images in Appendix S1 as a guide and following the schematic design, protoboard all electronic components in a PCB. Notice that the P‐channel MOSFET and the Power/Sense resistor are mounted on the heatsinks rather than on the PCB.
4. Using 22 AWG wire, solder the P‐channel MOSFET and the Power/Sense resistor to the PCB.
5. Using the included lead wires, solder the fan assembly and the COB‐LED holder to the PCB.

### Lamp assembly

1. From the GitHub repository, download the *Lamp.iam* assembly file as well as files for all individual parts (i.e., all **.ipt* files).
2. Visualize the lamp assembly using the AutoDesk Viewer online tool (https://viewer.autodesk.com/designviews).
3. By exploring the assembly with the online viewer, identify the placement of all components.
4. Solder 22 AWG wire leads to the power jack.
5. Install the power jack and cable gland in the lamp main body.
6. Solder the power leads to the PCB.
7. Install the P‐channel MOSFET and the Power/Sense resistor onto the TO‐220 heatsinks, using M3 × 6 mm screws. Make sure you use dielectric thermal pads and nylon washers to electrically isolate the heatsink from the electronic components.
8. Secure the TO‐220 heatsinks to the lamp main body using M2.5 screws.
9. Insert the multiconductor cable through the cable gland.
10. Connect the individual conductors to the PCB terminal block.
11. Connect the individual conductors on the other end of the cable to the D‐Sub standard connector.
12. Place the PCB into the slots inside the lamp main body.
13. Remove the fan from the CPU cooler heatsink.
14. Remove the clips to attach the fan frame to a computer motherboard.
15. Using a 3‐mm drill bit, drill a hole through the CPU cooler heatsink from top to bottom.
16. Place thermal paste onto the CPU cooler heatsink.
17. Secure the COB‐LED to the CPU cooler using the COB‐LED holder and 2.5 screws.
18. From the top of the CPU cooler heatsink, fill the hole drilled in step 14 with thermal paste.
19. Insert the temperature probe (NTC thermistor) in the hole filled with thermal paste so it touches the back plate of the COB‐LED array.
20. Use a piece of Kapton tape to secure the temperature sensor wires to the heatsink.
21. Place back the fan on top of the CPU cooler heatsink.
22. Make sure all wires are nicely arranged away from the COB‐LED.
23. Tighten the cable gland.
24. On the *Filter Holder_25.4 *mm part, place four pieces of 3‐mm‐wide, double‐sided tape into the four slots situated around the filter holding edge; Appendix S1C shows an image of the part after this step is completed.
25. Using a stereoscopic microscope and side illumination, identify the engineered side of the ED1‐S20 diffuser; the engineered side has a textured, sandy appearance (see Appendix S1D).
26. Using the stereoscopic microscope, place the ED1‐S20 diffuser into the filter holder part. Make sure that the engineered side is facing up and that the square pattern is aligned with one of the four slots situated around the filter holder edge. Appendix S1D shows an image of the part after this step is completed.
27. Place the retaining ring into the filter holder, making sure that the serrated side is facing towards the top of the ED1‐S20 diffuser.
28. Glue the retaining ring to the filter holder using super glue.
29. Place the filter holder onto the lamp main body, as shown in Appendix S1B.
30. Place a fan shim in its designated slots at each corner of the lamp main body, as shown in Appendix S1B.
31. Place the CPU cooler on top of the lamp main body.
32. Place the fan lid on top of the fan and secure it with M3 × 12 mm screws.
33. Insert the mirrors in their designated slots in the underpart of the lamp main body. It is recommended to secure the mirrors with a piece of folded paper inserted between the mirror and the lamp main body.
34. Using M3 × 16 mm screws, attach the lamp holder to the LI‐6800‐12A Clear‐top Leaf Chamber.
35. Place the lamp assembly on top of the lamp holder and secure it using M3 × 10 mm screws.
36. Connect the D‐Sub standard connector to the LI‐6800 *User I/O* port.
37. Connect the AC‐DC power supply to the lamp power jack.

### Microcontroller software installation

1. From the GitHub repository, download the *Microcontroller_Main.py* file.
2. Install the latest CircuitPython version in the Trinket M0 microcontroller following the manufacturer instructions (https://learn.adafruit.com/adafruit-trinket-m0-circuitpython-arduino/circuitpython).
3. Access the microcontroller CIRCUITPY drive following the manufacturer instructions (https://learn.adafruit.com/welcome-to-circuitpython/the-circuitpy-drive).
4. Delete the *code.py* file at the CIRCUITPY drive.
5. Rename the *Microcontroller_Main.py* file to *main.py* and upload it to the microcontroller CIRCUITPY drive.

### LI‐6800 software configuration

1. From the GitHub repository, download the *LI‐6800 Lamp Config*, *PAR_Ctrl.py*, *PAR_Ctrl_wFB.py*, and *A‐PPFD_Curve.py* files and copy them to a USB stick.
2. Insert the USB stick in the LI‐6800 console and navigate to *Tools > Manage Files*.
3. Select the option *Copy files to LI‐6800*.
4. Copy the *LI‐6800 Lamp Config* file to the configuration directory*/home/licor/configs*.
5. Copy the *PAR_Ctrl.py* and *PAR_Ctrl_wFB.py* files to the apps directory*/home/licor/apps*.
6. Navigate to *Start Up > Configuration* and select the *Load configurations* option from the drop‐down menu.
7. Select the configuration file you copied in step 4 and press the *Load* button.

## Appendix 2 Lamp PPFD output monitor and control calibration.

Note: This section provides step‐by‐step instructions to obtain the calibration coefficients needed to accurately monitor and control the lamp PPFD output. This section assumes that all building and configuration procedures described in Appendix 1 are completed. The procedure is done in four steps. First, the zero offset of the XTR110 chip is adjusted. Second, PPFD levels at various DAC1 control voltages are measured. Then, a new photodiode multiplier is calculated. The new multiplier is used to monitor PPFD using the internal LI‐6800‐12A leaf chamber photodiode. Finally, the parameters of a linear model are calculated. These parameters are needed to predict PPFD output based on COB‐LED heatsink temperature and DAC1 voltage. PPFD must be measured with an external device. Here we describe the procedures if using the LI‐COR Biosciences light meter (model LI‐250A) equipped with the quantum sensor (model LI‐190R Quantum); however, the procedure can easily be adapted if another model and/or brand is used instead.

### XTR110 zero offset adjustment

1. Turn OFF the LI‐6800.
2. Disconnect the lamp power supply.
3. Open the lamp enclosure and expose the trim pot potentiometer (R1 in the schematics).
4. Set a multimeter to measure DC current in the mA or μA range (μA is preferred).
5. Connect the multimeter in series with the negative lead coming from the COB‐LED to measure the COB‐LED current.
6. Cover the COB‐LED with a non‐transparent, heat‐resistant material (e.g., a metal end cap) to prevent light reaching the eyes of the user. It is strongly recommended to wear appropriate eye protection.
7. Ensure that all exposed circuit metal parts are not touching any other part.
8. Double‐check steps 6 and 7.
9. Connect the lamp power supply.
10. Turn ON the LI‐6800.
11. Navigate to the Console User I/O and turn ON the 5 V power supply (Power5).
12. Set DAC1 voltage to 0.005 V.
13. Measure the resulting current flowing through the COB‐LED; it should be in the order of 1000 μA.
14. Adjust the R1 trim pot until the COB‐LED current reaches exactly 1000 μA.
15. Set DAC1 voltage to 0 V.
16. Measure the resulting current flowing through the COB‐LED; it should be less than 16 μA; this leak current should disappear once the multimeter is not part of the circuit anymore. Further details on steps 12 to 16 are provided in the XTR110 datasheet (section “*Offset (zero) adjustment*”).
17. Turn OFF the LI‐6800.
18. Disconnect the lamp power supply.
19. Disconnect the multimeter.
20. Reconnect the negative COB‐LED lead as shown in the schematics.
21. Close the lamp enclosure.

### Taking needed measurements

1. Place the LI‐6800 console, the leaf chamber, and the attached lamp in a dark room, preferably with air conditioning.
2. If possible, set the room temperature at a setpoint similar to the temperature at which the lamp will be used.
3. Remove the leaf chamber aperture insert from the bottom side of the LI‐6800‐12A leaf chamber.
4. Remove the leaf temperature sensor from the bottom side of the LI‐6800‐12A leaf chamber.
5. Install the 3 × 3 cm leaf chamber aperture insert only in the upper side of the LI‐6800‐12A leaf chamber.
6. Place the quantum sensor beneath the leaf chamber aperture insert in such a way that the sensing element is at the leaf plane. Use a laboratory stand and clamp to securely fix the sensor in place.
7. Increase DAC1 voltage gradually, from 0 to 5 V, in increments of 0.25 V. At each step, record *Q*_*amb_in*_ and COB‐LED heatsink temperature, using the LI‐6800 console. At each step, also record the PPFD value measured by the quantum sensor installed in step 6, using an external light meter.
8. Repeat steps 5 to 7 using the 2 × 3 and 1 × 3 cm leaf chamber aperture inserts.
9. Repeat steps 5 to 8 at a different room temperature.
10. Prepare a data set in Excel with all measured data. Make sure to include the following variables as columns: DAC1 voltage, *Qamb_in*, PPFD measured by external sensor, COB‐LED heatsink temperature, and leaf chamber aperture insert area in cm^2^.

### Photodiode multiplier calibration

1. Obtain the original photodiode multiplier value from the leaf chamber memory by navigating to *Environment > Light > Ambient* in the LI‐6800 console.
2. Write down the original photodiode multiplier value and keep it.
3. From the measurements obtained during the previous subsection, filter out the data that has a saturated *Q*_*amb_in*_ value. Use the remaining data in the following steps.
4. Calculate the slope of the linear regression between the measured PPFD using the external quantum sensor (*x*‐axis) and *Q*_*amb_in*_ (*y*‐axis).
5. Calculate the new photodiode multiplier by dividing the original multiplier value by the slope calculated in the previous step.
6. In the LI‐6800 console, navigate to *Environment > Light > Ambient* and input the new photodiode multiplier.

### Lamp PPFD control model calibration

In this subsection, we provide detailed instructions to perform a multiple linear regression in SAS Studio (other software can be used, for example, R or Python).

1. Create an account in SAS OnDemand for Academics (https://www.sas.com/en_us/software/on-demand-for-academics.html).
2. Log in to SAS Studio.
3. Upload to SAS Studio the Excel file obtained in the “Taking needed measurements” subsection of Appendix 2 by navigating to *Server Files and Folders > Upload*.
4. Export the data to the *WORK* library folder as *IMPORT* library by navigating to *Tasks and Utilities > Utilities > Import Data*.
5. Start configuring a multiple linear regression task by navigating to *Task and Utilities > Tasks > Linear Models > Linear Regression*.
6. In the *DATA* tab, select the *WORK.IMPORT* library in the *Data* field.
7. Select the PPFD measured by the external quantum sensor as *Dependent variable*.
8. Select the DAC1, COB‐LED heatsink temperature, and aperture insert area as *Continuous variables*.
9. In the *MODEL* tab, click on the *Edit* button and add the model effects as outlined by Eq. 1, without changing the estimates order. Make sure to deselect the *use intercept* option.
10. Run the task.
11. If all goes well, the *r* ^2^ value for the model should be 1 or very close to 1, and the *P* value for the model in the ANOVA table should be lower than 0.001.
12. Write down the four parameter estimates.
13. In the LI‐6800 console, navigate to *Constants > User* and click on the *Edit/Reorder* option.
14. Select the user variable named *Lamp_PAR_DAC1_Ctrl* and click on the *Edit* button.
15. Replace the parameters named a1 to a4 with the parameters noted in step 12. Make sure that the parameter order matches the order of both Eq. 1 and the model equation entered in step 9.
16. Save the changes and click on the *Edit/Reorder* option to prevent the user from accidentally changing user variables and constants.
17. Navigate to *Start up > Configuration* and select the *Save configurations* option from the drop‐down menu.
18. Deselect all available settings to save, except for the user‐defined constants (*UsrDefCon*).
19. Save the changes to the configuration file.

## Appendix 3 Temperature control calibration.

Note: This section provides step‐by‐step instructions to calibrate the control voltage over the TEMP_CTRL pin. This section assumes that all building and configuration procedures described in Appendix 1 are completed. A multimeter is required for this calibration.

1. From the GitHub repository, download the *Zenner Diode Calibration_Data Template.xlsx* and *Curve_Fit_Zener_Diode.py* files. Place the files in the same directory.
2. Get the multimeter ready to measure the voltage between the TEMP_CTRL and ground pins at the microcontroller side. The point of measurement should be between the Zener diode and the microcontroller, and as close as possible to the microcontroller.
3. At the LI‐6800 console, increase DAC2 voltage from 0 to 5 V in steps of 0.2 V. At each step, record the DAC2 and TEMP_CTRL voltages in the Excel file downloaded in step 1.
4. Execute the Python script downloaded in step 1.
5. Write down the estimated coefficients.
6. In the LI‐6800 console, navigate to *Constants > User* and click on the *Edit/Reorder* option.
7. Select the user variable named *Lamp_T_DAC2_V_Ctrl* and click on the *Edit* button.
8. Replace the parameters named a1 to a4 with the parameters noted in step 5. Make sure that the parameter order matches Eq. 3.
9. Save the changes and click on the *Edit/Reorder* option to prevent the user from accidentally changing user variables and constants.
10. Navigate to *Start up > Configuration* and select the *Save configurations* option from the drop‐down menu.
11. Deselect all available settings to save except for the user‐defined constants (*UsrDefCon*).
12. Save the changes to the configuration file.

## Appendix 4 Four example uses of the lamp.

Note: This section assumes that all building and configuration procedures described in Appendix 1 are completed.

### Direct voltage control of the lamp

Direct voltage control over the lamp is intended only for development and calibration procedures.

1. Navigate to *Start Up > Configuration* and select the *Load configurations* option from the drop‐down menu.
2. Select the lamp configuration file and press the *Load* button.
3. Navigate to *Start Up > Peripheral Setup > Console User I/O*.
4. Make sure the *Power5* supply is on, as it is needed to power the temperature sensor and microcontroller.
5. At the *DAC1* field, enter the desired LED_CTRL voltage and press the *Done* button.
6. Navigate to *Measurements > Grid 4*.
7. Observe the user‐defined variables and constants related to the lamp. Notice that the lamp PPFD changes (*Qin* variable) as the COB‐LED heatsink temperature changes (*Lamp_T* user variable).

### Lamp PPFD control without feedback

1. Navigate to *Start Up > Configuration* and select the *Load configurations* option from the drop‐down menu.
2. Select the lamp configuration file and press the *Load* button.
3. Navigate to *Programs* > *BP Builder > Open/New*.
4. Select the *PAR_Ctrl.py* file and press the *Start BP* button.
5. Navigate to *Constants > User*.
6. Input the desired PPFD in the *Lamp_PAR_Ctrl* user constant field.
7. Navigate to *Measurements > Grid 4*.
8. Observe the user‐defined variables and constants related to the lamp. Notice that the *Lamp_Qamb_in_Factor* user constant will not change, as the *PAR_Ctrl.py* script uses no feedback.
9. When you finish your experiment, navigate to *Programs* > *BP Monitor*.
10. From the table listing all BPs running, select the *PAR_Ctrl.py* BP.
11. Press the *Cancel* button to stop the BP.

### Lamp PPFD control with feedback

1. Navigate to *Start Up > Configuration* and select the *Load configurations* option from the drop‐down menu.
2. Select the lamp configuration file and press the *Load* button.
3. Navigate to *Programs* > *BP Builder > Open/New*.
4. Select the *PAR_Ctrl_wFB.py* file and press the *Start BP* button.
5. Navigate to *Constants > User*.
6. Input the desired PPFD in the *Lamp_PAR_Ctrl* user constant field.
7. Navigate to *Measurements > Grid 4*.
8. Observe the *Lamp_Qamb_in_Factor*, which indicates the level of feedback that the *PAR_Ctrl_wFB.py* script is applying.
9. When you finish your experiment, navigate to *Programs* > *BP Monitor*.
10. From the table listing all BPs running, select the *PAR_Ctrl_wFB.py* BP.
11. Press the *Cancel* button to stop the BP.

### Manual A‐PPFD curve

1. Navigate to *Start Up > Chamber Setup* and select the installed leaf chamber aperture insert.
2. Navigate to *Start Up > Configuration* and select the *Load configurations* option from the drop‐down menu.
3. Select the lamp configuration file and press the *Load* button.
4. Navigate to *Start up > Warmup Tests*.
5. From the drop‐down menu, select the option *Warmup Tests* and press the *Start* button.
6. If the warmup tests find no issues, continue to the next step; otherwise correct any issues before progressing.
7. Navigate to the *Environment* tab and set the desired air flow, VPD, chamber CO_2_ concentration, fan speed, and leaf temperature.
8. Clamp the leaf to be measured.
9. Navigate to *Log Setup > Stability* and define the stability definition you wish to use (see section 4 of the LI‐6800 manual for details [LI‐COR Biosciences, 2021]).
10. Navigate to *Log Setup > Matching Options* and define the automatic matching schedule that you wish to use. It is recommended to match at least every 30 min and/or after a change in CO_2_ concentration (see section 4 of the LI‐6800 manual for details [LI‐COR Biosciences, 2021]).
11. Navigate to *Log Setup > Open a Log File* and create a new log file by pressing the *New File* button.
12. Navigate to *Programs* > *BP Builder > Open/New*.
13. Select the *PAR_Ctrl.py* file and press the *Start BP* button.
14. Navigate to *Constants > User*.
15. Input the desired PPFD in the *Lamp_PAR_Ctrl* user constant field. Within 10 s, the lamp should automatically adjust to the desired PPFD.
16. Navigate to the *Measurements* tab and monitor the leaf response to the selected PPFD.
17. Wait for stability. When stability is reached, press the *Log* button.
18. Repeat steps 14 to 17 until you finish measuring all PPFD steps in the A‐PPFD curve.
19. Navigate to *Log Setup > Logging to* and press the *Close Log* button.
20. Navigate to *Programs* > *BP Monitor*.
21. From the table listing all BPs running, select the *PAR_Ctrl.py* BP.
22. Press the *Cancel* button to stop the BP.

### Programmatic A‐PPFD curve

1. Navigate to *Start Up > Chamber Setup* and select the installed leaf chamber aperture insert.
2. Navigate to *Start Up > Configuration* and select the *Load configurations* option from the drop‐down menu.
3. Select the lamp configuration file and press the *Load* button.
4. Navigate to *Start up > Warmup Tests*.
5. From the drop‐down menu, select the option *Warmup Tests* and press the *Start* button.
6. If the warmup tests find no issues, continue to the next step; otherwise correct any issues before progressing.
7. Navigate to *Programs* > *BP Builder > Open/New*.
8. Select the *A‐PPFD_Curve.py* file and press the *Open BP* button.
9. Navigate to *Programs* > *BP Builder > Set*.
10. Expand the groups *Settings > Def. environmental settings*.
11. Adjust all environmental settings that the plant material/experiment needs. Here you define the flow, VPD, CO_2_ concentration, fan speed, leaf temperature, and chamber differential pressure to be set during the experiment. Notice that there are three variables for flow (*Low_flow_set*, *Medium_flow_set*, and *High_Flow_set*); this is because the script uses different flow sets at different PPFD levels. The higher the PPFD, the higher the script sets the flow. This is to ensure maximum signal‐to‐noise ratio while keeping a ΔCO_2_ lower than 30 μmol∙m^−2^∙s^−1^ (as recommended by the equipment manufacturer). The PPFD thresholds defining each flow level are set in step 15.
12. Expand the groups *Settings* > *A‐PPFD*.
13. Adjust the *PAR_levels* variable according to the plant material/experiment needs. The values you enter here are the PPFD values that will be used during the A‐PPFD curve. The script will use the values in the order in which they are entered.
14. Adjust the *PAR_wait* variable according to the plant material/experiment needs. The values you enter here are the minimum waiting time (min) that script will keep each PPFD level. The order and number of items in the list must match the values entered in step 13.
15. Adjust the *Low_flow_PAR_end* and *High_flow_PAR_start* variables according to the plant material/experiment needs. These two variables are PPFD threshold levels defining three flow sets (see step 11 for explanation).
16. Press the *Save* button to skip all environment settings adjustments next time you use this script.
17. Clamp the leaf to be measured.
18. Navigate to *Log Setup > Stability* and define the stability definition you wish to use (see section 4 of the LI‐6800 manual for details [LI‐COR Biosciences, 2021]).
19. Navigate to *Log Setup > Matching Options* and define the desired automatic matching schedule. It is recommended to match at least every 30 min and/or after a change in CO_2_ concentration (see section 4 of the LI‐6800 manual for details [LI‐COR Biosciences, 2021]).
20. Navigate to *Log Setup > Open a Log File* and create a new log file by pressing the *New File* button.
21. Navigate to *Programs* > *BP Builder > Open/New*.
22. Select the script file you saved in step 16 and press the *Start BP* button.
23. Navigate to *Programs* > *BP Monitor* to monitor the two scripts that are running the A‐PPFD curve. Notice that the main script automatically runs the *PAR_Ctrl.py* script.
24. Navigate to the *Measurements* tab and monitor the leaf responses to the PPFD levels.
25. When the script finishes the A‐PPFD curve, navigate to *Log Setup > Logging to* and press the *Close Log* button.
26. Navigate to *Programs* > *BP Monitor*.
27. From the table listing all BPs running, select the *PAR_Ctrl.py* BP.
28. Press the *Cancel* button to stop the BP.
