# Supplementary material for: An open‐source LED lamp for use with the LI‐6800 photosynthesis system
Source: Appl Plant Sci. 2024 Oct 25;13(1):e11622. doi: 10.1002/aps3.11622 (PMC11788902; doi:10.1002/aps3.11622)
Supplement: Supplementary file 1 — Appendix S1. Mechanical details of a lamp for the LI‐6800 photosynthesis system. Appendix S2. Spectral effects of the ED1‐S20 diffuser (A) and Propafilm C leaf chamber covering film (B). Appendix S3. Correlation between irradiance measured by an external quantum sensor and the internal photodiode. Appendix S4. Cosine corrections for the lamp without (A) and with the ED1‐S20 diffuser installed (B). Appendix S5. Photosynthetic photon flux density (PPFD) distributions at the leaf plane, using the 3 × 3 cm, 2 × 3 cm, and 1 × 3 cm aperture inserts, without the diffuser installed. Appendix S6. Photosynthetic photon flux density (PPFD) distributions at the leaf plane, using the 3 × 3 cm, 2 × 3 cm, and 1 × 3 cm aperture inserts, with the ED1‐S20 diffuser installed. Appendix S7. Table summarizing the lamp photosynthetic photon flux density (PPFD) uniformity scans. Appendix S8. Effect of the Zenner diode protection. Appendix S9. Attainable COB‐LED heatsink temperature setpoint. Appendix S10. Attainable photosynthetic photon flux density (PPFD) without the diffuser (A) and using the ED1‐S20 diffuser (B). Appendix S11. Technical specifications for the lamp described in this paper, alongside two commercially available lamps from LI‐COR Biosciences (Lincoln, Nebraska, USA). [file APS3-13-e11622-s001.pdf]

Vélez-Ramírez et al.—Applications in Plant Sciences 2024—Data Supplement S1
DOI 10.1002/aps3.11622

Supporting information for "An open-source LED lamp for use with the LI-6800 photosynthesis system"

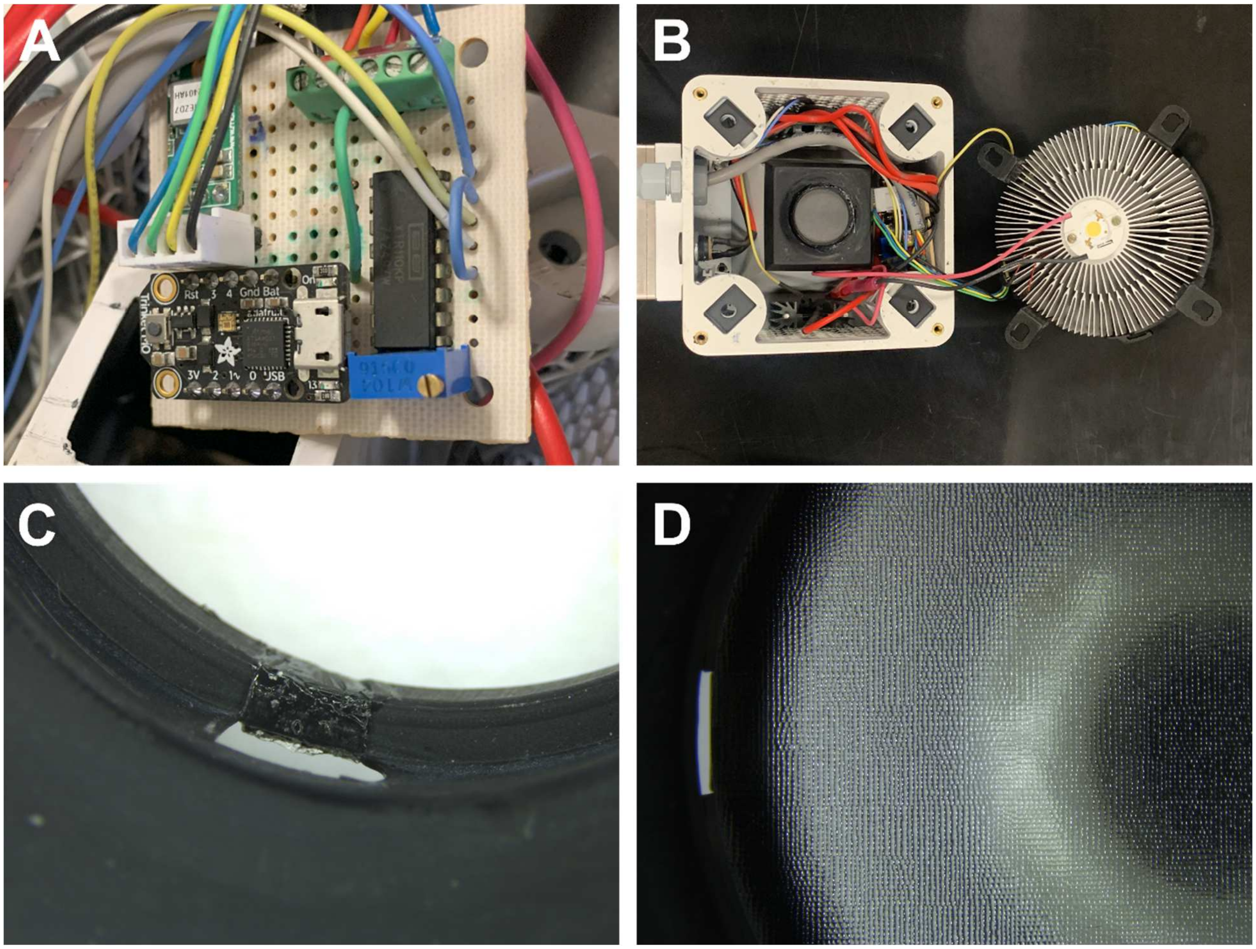

Appendix S1. Mechanical details of a lamp for the LI-6800 photosynthesis system. In panel (**A**), a protoboard with the DC-DC voltage converter (top left), the XTR110 chip (right), the microcontroller (bottom left) and the tri pod (bottom right). In panel (**B**), details of the ED1-S20 diffuser mounted on the Filter Holder. In panel (**C**), 3-mm-wide, double-sided tape placed in one of the four slots situated around the filter holding edge. In panel (**D**), the ED1-S20 diffuser square pattern is aligned with one of the four slots situated around the filter holding edge.

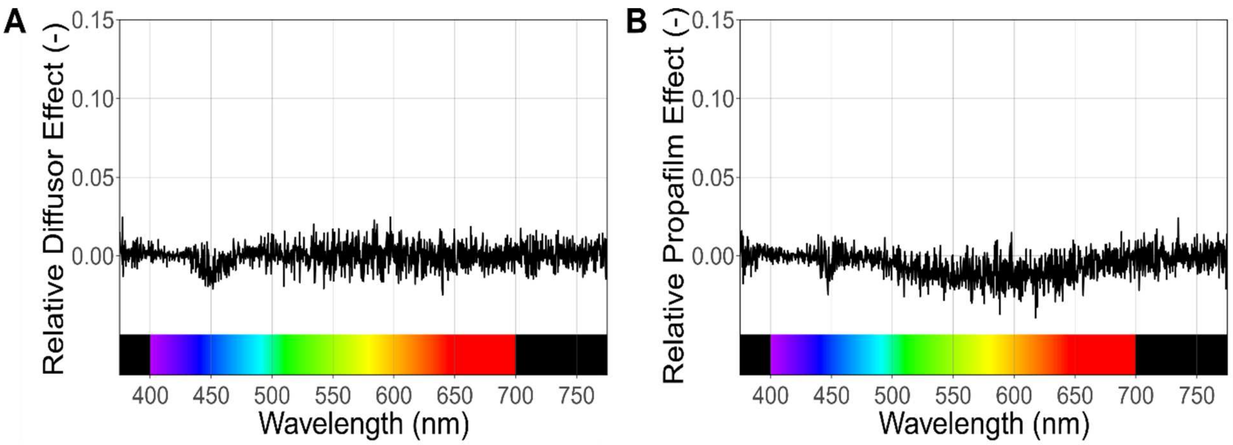

Appendix S2. Spectral effects of the ED1-S20 diffuser (Panel A) and Propafilm C leaf chamber covering film (LI-COR part #250-01885) (Panel B). Values were calculated as the relative spectrum of the unfiltered COB-LED light subtracted by the relative spectrum of the filtered light.

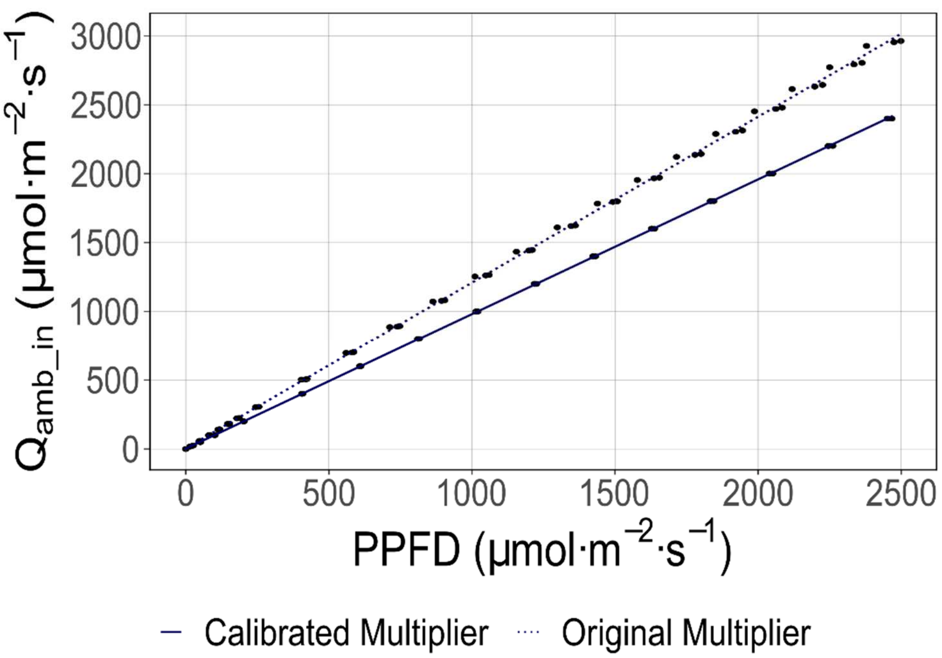

Appendix S3. Correlation between irradiance measured by an external quantum sensor (PPFD, *x*-axis) and the internal photodiode (Q_{amb_in}, *y*-axis). The dotted line indicates the correlation before multiplier calibration, and the solid line depicts the calibration after the multiplier calibration.

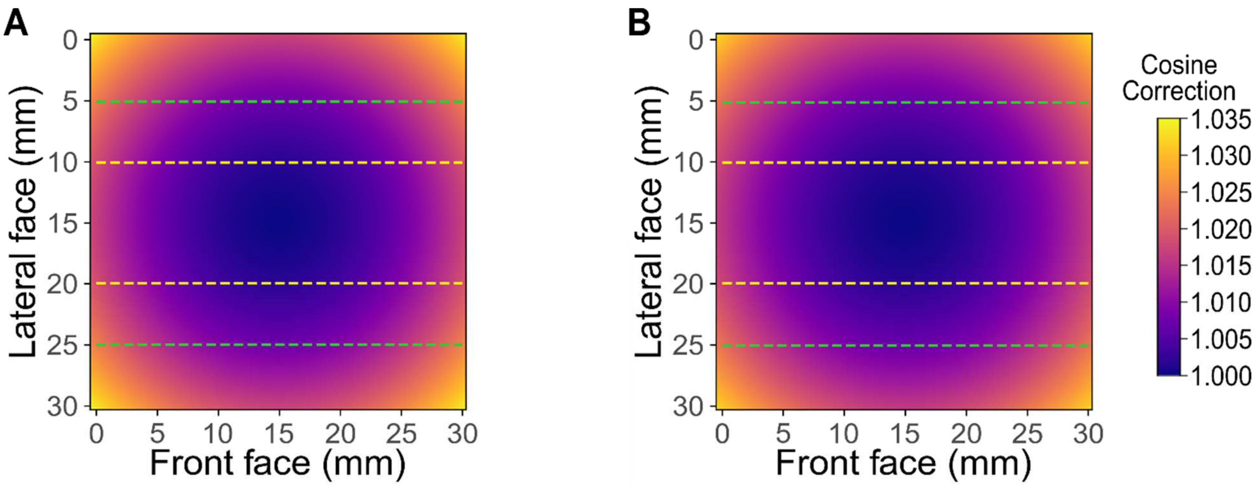

Appendix S4. Cosine corrections for the lamp without (Panel A) and with the ED1-S20 diffuser installed (Panel B). Both panels depict the area corresponding to the 3 × 3 insert. The same cosine correction is applied to the three inserts, with the difference that the small inserts (2 × 3 and 1 × 3) are delimited by the horizontal dotted lines. The area between the yellow dotted lines represents the cosine correction for the 1 × 3 insert, and the area between the dotted green line represents the area for the 2 × 3 insert. A margin of 0.4 mm is included in both panels.

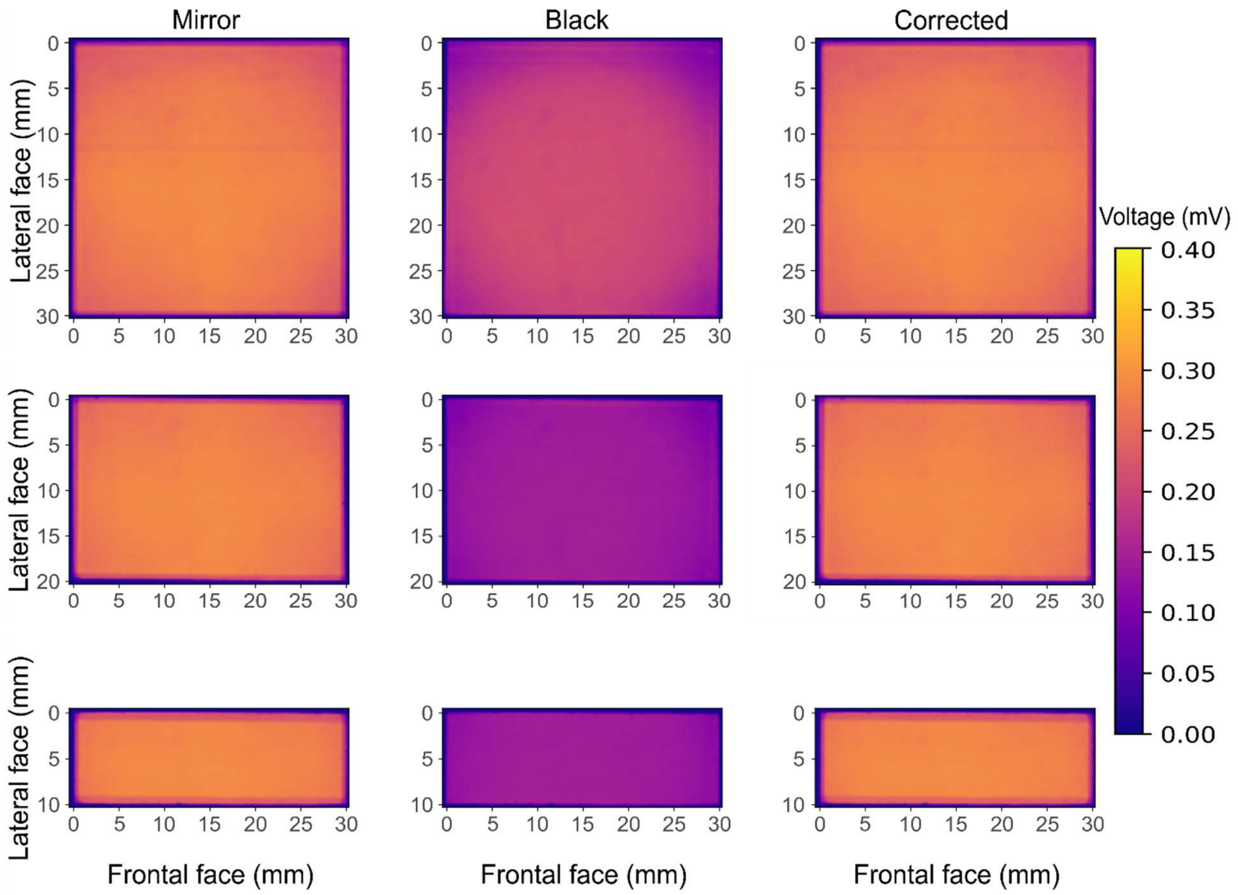

Appendix S5. Photosynthetic photon flux density (PPFD) distributions at the leaf plane, using the 3 × 3 cm, 2 × 3 cm, and 1 × 3 cm aperture inserts, without the diffuser installed. The images in the first column (labeled "mirror") represent the raw homogeneity measurements with the lamp assembled as described in Appendix S1. The middle column (labeled "black") shows the raw homogeneity measurements taken with the mirrors covered by black-painted sheets. The last column (labeled "corrected") displays the corrected homogeneity measurements, calculated as described by Equation 5. A margin of 0.4 mm is included.

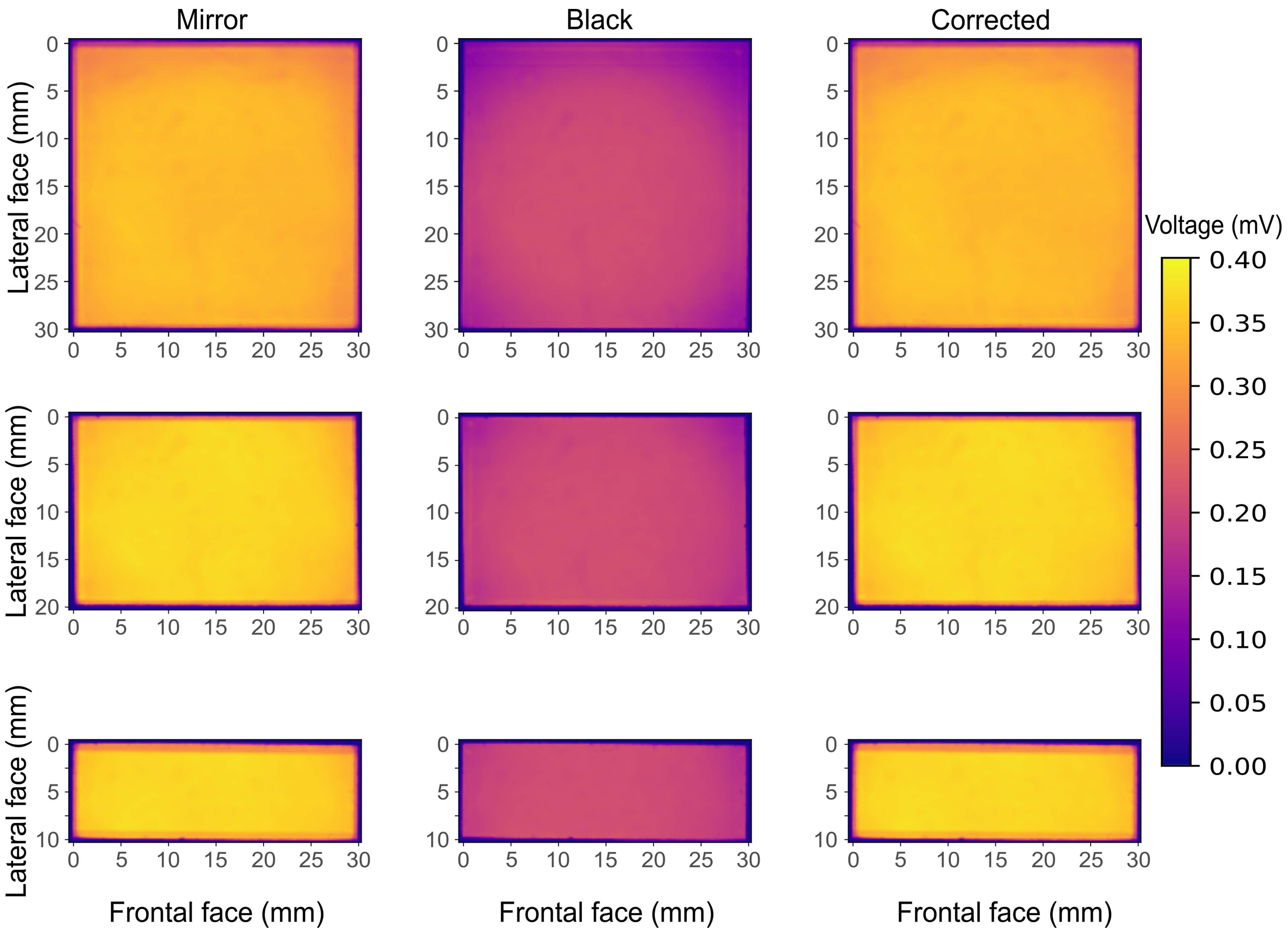

Appendix S6. Photosynthetic photon flux density (PPFD) distributions at the leaf plane, using the 3 × 3 cm, 2 × 3 cm, and 1 × 3 cm aperture inserts, with the ED1-S20 diffuser installed. The images in the first column (labeled "mirror") represent the raw homogeneity measurements with the lamp assembled as described in Appendix S1. The middle column (labeled "black") shows the raw homogeneity measurements taken with the mirrors covered by black-painted sheets. The last column (labeled "corrected") displays the corrected homogeneity measurements, calculated as described by Equation 5. A margin of 0.4 mm is included.

Appendix S7. Table summarizing the lamp photosynthetic photon flux density (PPFD) uniformity scans. Measurements are shown as raw readings from the photodiode in millivolts (mV).

Metric	Without diffuser			With diffuser ED1-S20		
	3x3 insert	2x3 insert	1x3 insert	3x3 insert	2x3 insert	1x3 insert
**Mean (mV)**	262.11	266.06	267.08	326.71	351.71	349.86
**Median (mV)**	271.08	275.84	280.42	337.81	365.12	365.95
**SD (mV)**	30.75	34.24	35.73	31.59	42.51	41.02
**Min (mV)**	11.43	10.17	8.88	65.64	10.03	9.18
**Max (mV)**	292.68	292.69	292.66	358.07	378.61	379.26
**CV at 90% area (%)**	5.4	3.53	6.4	4.01	2.47	5.85
**Area with CV lower than 10% (%)**	98	100	97	100	100	97

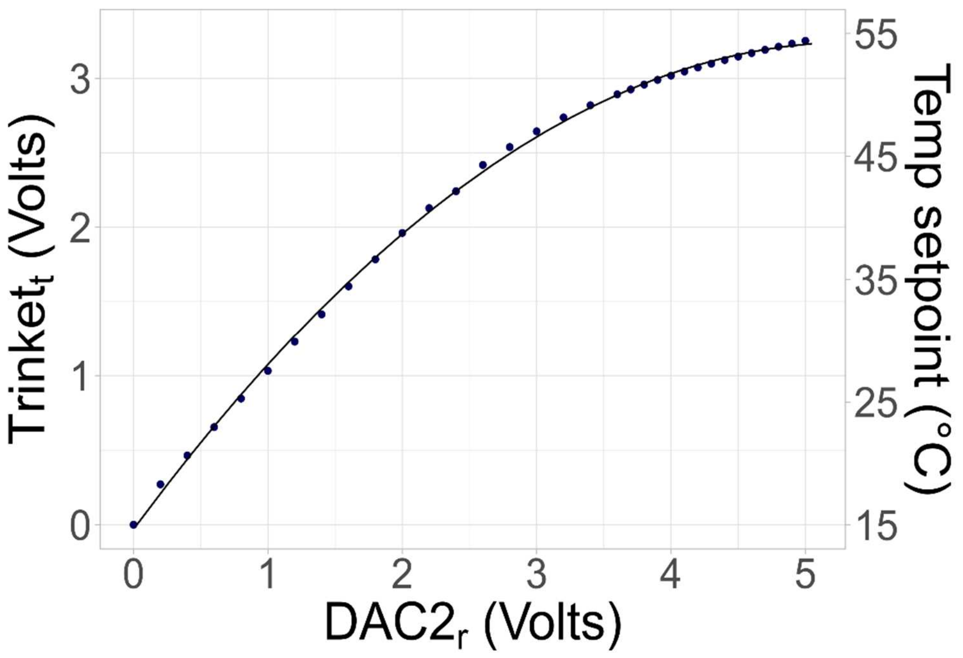

Appendix S8. Effect of the Zenner diode protection. The *x*-axis depicts the required voltage at the LI-6800 User I/O DAC2 channel ($DAC2_r$) that is needed to achieve the target voltage at the Trinket M0 microcontroller side ($Trinket_t$, primary *y*-axis). Dots represent measurements and the line is a regression according to Eq. 3. The second *y*-axis shows the resulting COB-LED heatsink temperature setpoint according to Eq. 4.

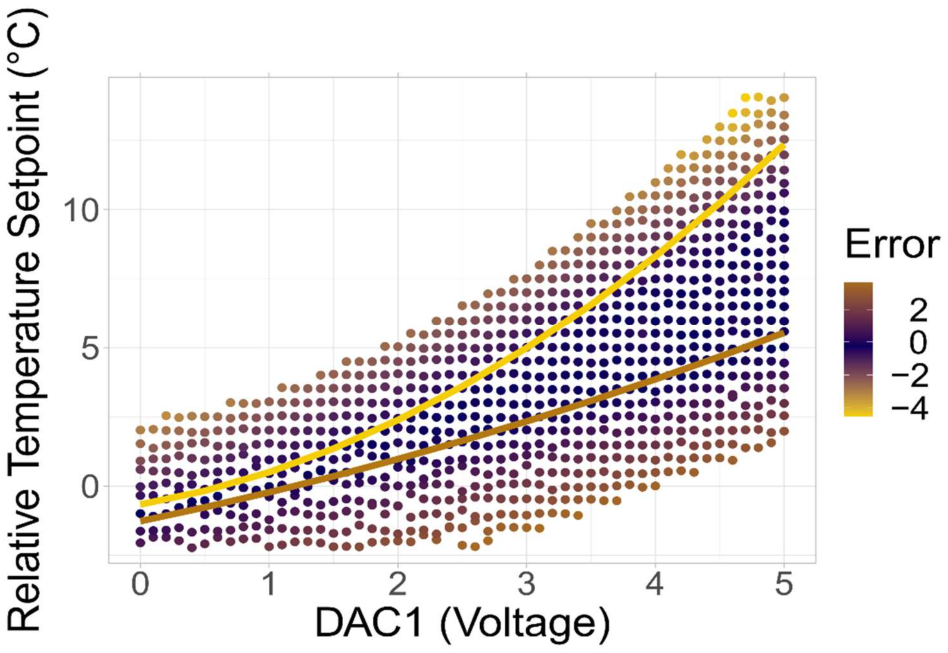

Appendix S9. Attainable COB-LED heatsink temperature setpoint. The *x*-axis shows the voltage at the LI-6800 User I/O DAC1 channel, which controls LED current. That is, at a higher DAC1 voltage, the current flowing though the COB-LED, the emitted irradiance, and the generated heat are larger. The *y*-axis depicts the COB-LED heatsink temperature setpoint, relative to air temperature. The dots depict 884 tested combinations of COB-LED intensity and heatsink temperature setpoint. The color scale indicates the deviation between the measured COB-LED heatsink temperature and its setpoint (Error in °C).

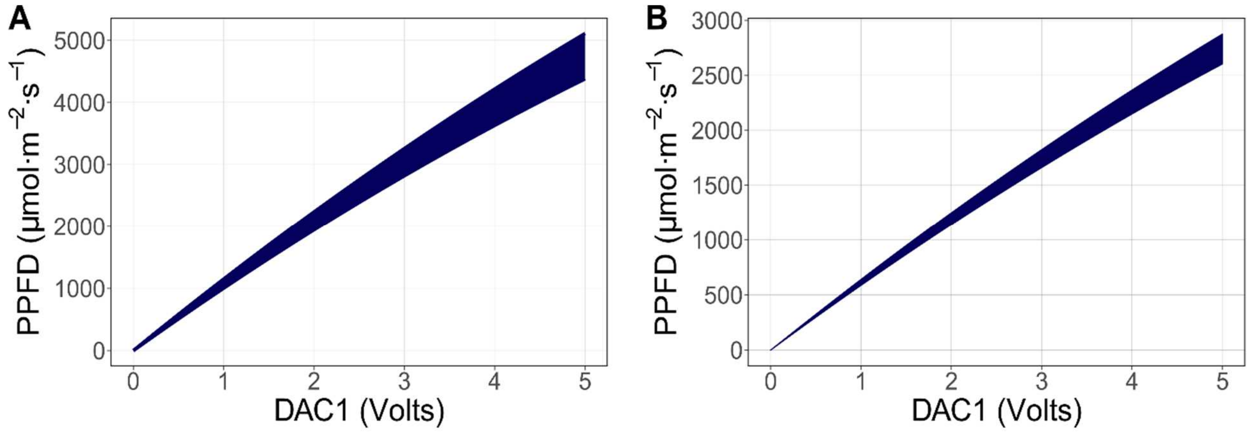

Appendix S10. Attainable photosynthetic photon flux density (PPFD) without the diffuser (Panel A) and using the ED1-S20 diffuser (Panel B). At any given DAC1 voltage, the shaded area indicates the attainable PPFD when the heatsink temperature is between 5 and 80°C.

Appendix S11. Technical specifications for the lamp described in this paper, compared to two commercially available lamps from LI-COR Biosciences (Lincoln, Nebraska, USA).

**Specification**	**Lamp described in this paper**		**Li-Cor Biosciences Small Light Source (LI-6800-02)**	**Li-Cor Biosciences Large Light source (LI-6800-03)**
	**Without diffuser**	**With ED1-S20 diffuser**		
**Total Output Range (μmol·m^{2}·s^{-1})**	0 – >4250	0 – >2500	0 – >2000	0 – >2500
**Blue Output Range (μmol·m^{2}·s^{-1})**	NA	NA	0 – >400	0 – > 2000
**Green Output Range (μmol·m^{2}·s^{-1})**	NA	NA	NA	0 – >1000
**Red Output Range (μmol·m^{2}·s^{-1})**	NA	NA	0 – >1600	0 – >2400
**White Output Range (μmol·m^{2}·s^{-1})**	0 – >4250	0 – >2500	NA	0 – > 1500
**Blue Peak Wavelength (nm)**	NA	NA	453	453
**Green Peak Wavelength (nm)**	NA	NA	NA	523
**Red Peak Wavelength (nm)**	NA	NA	660	660
**White Color Temperature (K)**	6500	6500	NA	4000
**Red to far-red ratio**	7.43	7.43	NA	Not disclosed

**Phytochrome photostationary state (PSS)** (Sager et al., 1988)	0.845	0.845	Not disclosed	Not disclosed
**Uniformity (CV%)**	±5.4% over 90% of the aperture	±4% over 90% of the aperture	±10 % over 90% of the aperture	±10 % over 90% of the aperture
**Power Consumption at 2000 μmol·m^{2}·s^{-1} PPFD (W)**	10.99	18.67	<5	15
**Operating Temperature Range (°C)**	0 – 50	0 – 50	0 – 50	0 – 50
**Operating Relative Humidity Range (%)**	Noncondensing	Noncondensing	Not reported	0 – 85
**Size L x W x H (cm)**	12.1 x 11 x 13.3	12.1 x 11 x 13.8	6.6 x 59 x 5.8	11.7 x 11 x 13
**Weight (g)**	969	1002	210	540
**Cost as of 2024 (USD)**	266	405	> 7100	> 7000
